# Human Surfactant Protein SP-A1 and SP-A2 Variants Differentially Affect the Alveolar Microenvironment, Surfactant Structure, Regulation and Function of the Alveolar Macrophage, and Animal and Human Survival Under Various Conditions

**DOI:** 10.3389/fimmu.2021.681639

**Published:** 2021-08-17

**Authors:** Joanna Floros, Nithyananda Thorenoor, Nikolaos Tsotakos, David S. Phelps

**Affiliations:** ^1^Center for Host Defense, Inflammation, and Lung Disease (CHILD) Research, Department of Pediatrics, The Pennsylvania State University College of Medicine, Hershey, PA, United States; ^2^Department of Obstetrics & Gynecology, The Pennsylvania State University College of Medicine, Hershey, PA, United States; ^3^Department of Biochemistry & Molecular Biology, The Pennsylvania State University College of Medicine, Hershey, PA, United States; ^4^School of Science, Engineering, and Technology, The Pennsylvania State University, Harrisburg, PA, United States

**Keywords:** alveolar macrophage, SFTPA1, SFTPA2, SP-A1, SP-A2

## Abstract

The human innate host defense molecules, SP-A1 and SP-A2 variants, differentially affect survival after infection in mice and in lung transplant patients. SP-A interacts with the sentinel innate immune cell in the alveolus, the alveolar macrophage (AM), and modulates its function and regulation. SP-A also plays a role in pulmonary surfactant-related aspects, including surfactant structure and reorganization. For most (if not all) pulmonary diseases there is a dysregulation of host defense and inflammatory processes and/or surfactant dysfunction or deficiency. Because SP-A plays a role in both of these general processes where one or both may become aberrant in pulmonary disease, SP-A stands to be an important molecule in health and disease. In humans (unlike in rodents) SP-A is encoded by two genes (*SFTPA1* and *SFTPA2*) and each has been identified with extensive genetic and epigenetic complexity. In this review, we focus on functional, structural, and regulatory differences between the two SP-A gene-specific products, SP-A1 and SP-A2, and among their corresponding variants. We discuss the differential impact of these variants on the surfactant structure, the alveolar microenvironment, the regulation of epithelial type II miRNome, the regulation and function of the AM, the overall survival of the organism after infection, and others. Although there have been a number of reviews on SP-A, this is the first review that provides such a comprehensive account of the differences between human SP-A1 and SP-A2.

## 1. Introduction

**Figure 1:**
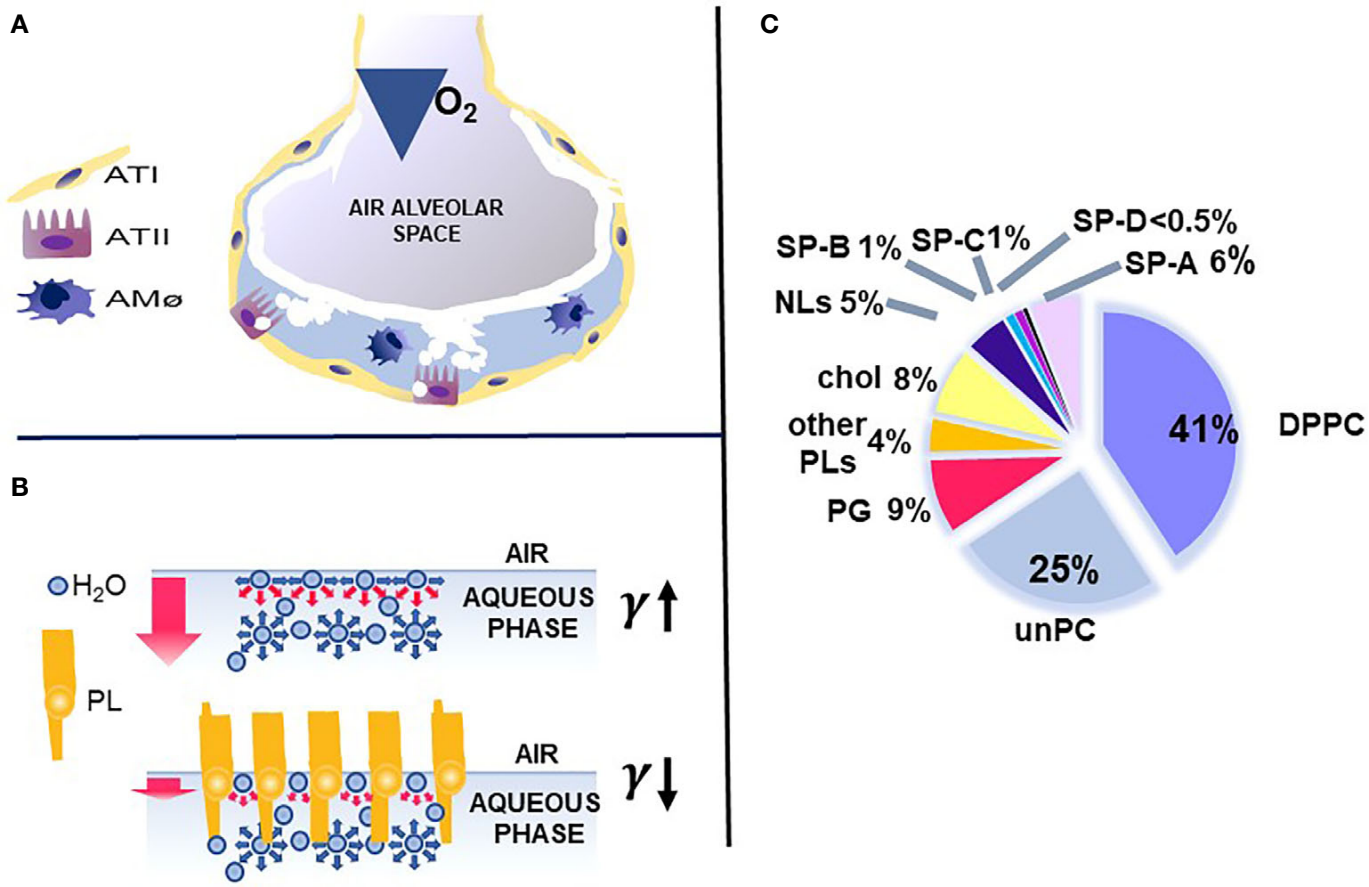
Alveolus, surfactant function, and surfactant composition. **(A)** depicts a cartoon of the alveolus, which is lined by epithelial type II and type I cells with the alveolar macrophage in the hypophase. In **(B)** the top air/aqueous interface lacks surfactant phospholipids and this leads to an increase in surface tension (γ). In the presence of phospholipids, the surface tension (γ) is reduced (bottom panel) and this prevents alveolar collapse. **(C)** depicts the lipid and protein composition of pulmonary surfactant. Courtesy of Dr. Jesus Perez-Gil (1).

The key function of the lung is the exchange of O_2_ and CO_2_, which is vital for the survival of the organism. This function occurs in the distal lung air spaces called alveoli. The alveolus is lined by epithelial cells, the type I cells, constituting the barrier for the CO_2_/O_2_ exchange and the type II cells, the cellular site of surfactant production. The alveoli are lined by a thin liquid layer, the hypophase (**Figure 1A**). Pulmonary surfactant, a lipoprotein complex, is found at the air-liquid interface of the alveolus, as well as in the hypophase, and is essential for life. By lowering surface tension at the air-liquid interface of the alveolus, surfactant prevents alveolar collapse at low lung volumes (**Figure 1B**).

**Figure 2:**
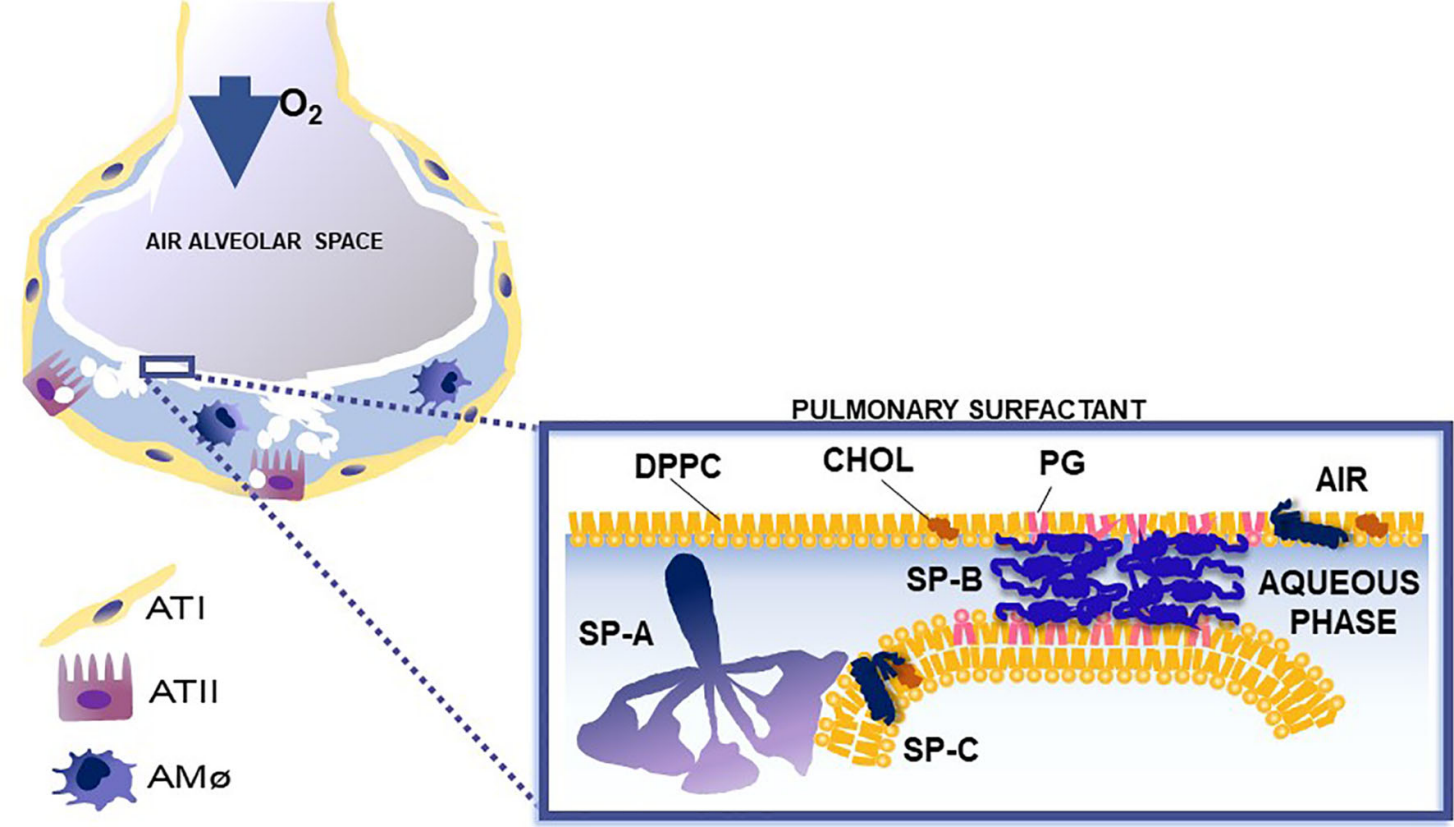
This cartoon depicts the role of surfactant proteins (SP-A, SP-B, and SP-C) in the biophysical properties of surfactant. Courtesy of Dr. Jesus Perez-Gil (1).

Pulmonary surfactant consists of lipids, primarily phospholipids, and non-serum-derived proteins, SP-A, SP-B, and SP-C (**Figure 1C**). A fourth protein SP-D co-isolates with surfactant and is grouped with the other three surfactant proteins, although it is not known to play a role in any surfactant-related functions, such as surface tension reduction or surfactant structure (2) (**Figure 2**). In general, SP-B and SP-C, both hydrophobic proteins, are primarily involved in surfactant-related functions, and SP-A and SP-D, both hydrophilic proteins, play a role in innate immune host defense, although SP-A does contribute to various aspects of surfactant structure, and function, as depicted, in part, in **Figure 2**. Both SP-A and SP-D belong to the collectin family. SP-D is present on the same genetic locus as SP-A1, SP-A2 (3), and mannose-binding lectin (MBL) (4, 5), is secreted by pulmonary type II epithelial cells, and it may play a role in surfactant homeostasis (6, 7)

Pulmonary surfactant consists of a monomolecular surface film at the air/liquid interface of the alveolus that is directly responsible for surface tension reduction, as well as a surfactant reservoir in the hypophase (1, 8–11). In the course of a breath (compression and expansion) the surface area of the alveolus undergoes significant change. Pulmonary surfactant is reorganized and an interconnection is formed and maintained between the two associated parts of surfactant (the surface film and the reservoir). SP-B and SP-C are essential for surfactant multilayer connection and bringing lipids to the air-liquid interface. SP-A, *via* its ability to bind phospholipids (dipalmitoyl phosphatidylcholine) may contribute to this (12–21). SP-A1 (see below) minimizes hysteresis during the respiratory cycle and is more efficient in facilitating lipid membrane reorganization and preventing surfactant inhibition by serum proteins compared to SP-A2 (22).

The surfactant proteins, SP-B, SP-C, and SP-D, are each encoded by a single gene (2) but SP-A, the focus of this review, in humans and primates (unlike rodents) is encoded by two genes, *SFTPA1* and *SFTPA2* (3, 5), and their respective gene products in humans are SP-A1 and SP-A2. The two genes are distinct but share a high degree of sequence similarity (23). The human SP-A locus is mapped on chromosome 10q22.3 (24), and consists of two functional genes, *SFTPA1* and *SFTPA2*, in opposite transcriptional orientation (3, 5). This locus also includes two pseudogenes, the *MBL3P* (mannose-binding lectin family member 3 pseudogene) (25) and *SFTPA3P* (surfactant protein A3 pseudogene (3, 5, 26). Each SP-A gene consists of four coding regions and a number of untranslated exons at the 5’ and one untranslated region at 3’ (27). A number of variants have been identified and characterized for each functional gene that may differentially affect regulation and/or function of alveolar cells, or the structure of surfactant films. These include variants resulting from alternative splicing events and variable translation initiation sites in the 5’-UTR (28–30), as well as isoforms with different signal peptide cleavage sites (23). Some of these have been shown to have an impact on translation efficiency and mRNA stability (31, 32). The 3’UTR also plays a role in SP-A regulation (33, 34). However, the differential regulation of these genes is discussed in detail in a review under preparation.

**Figure 3:**
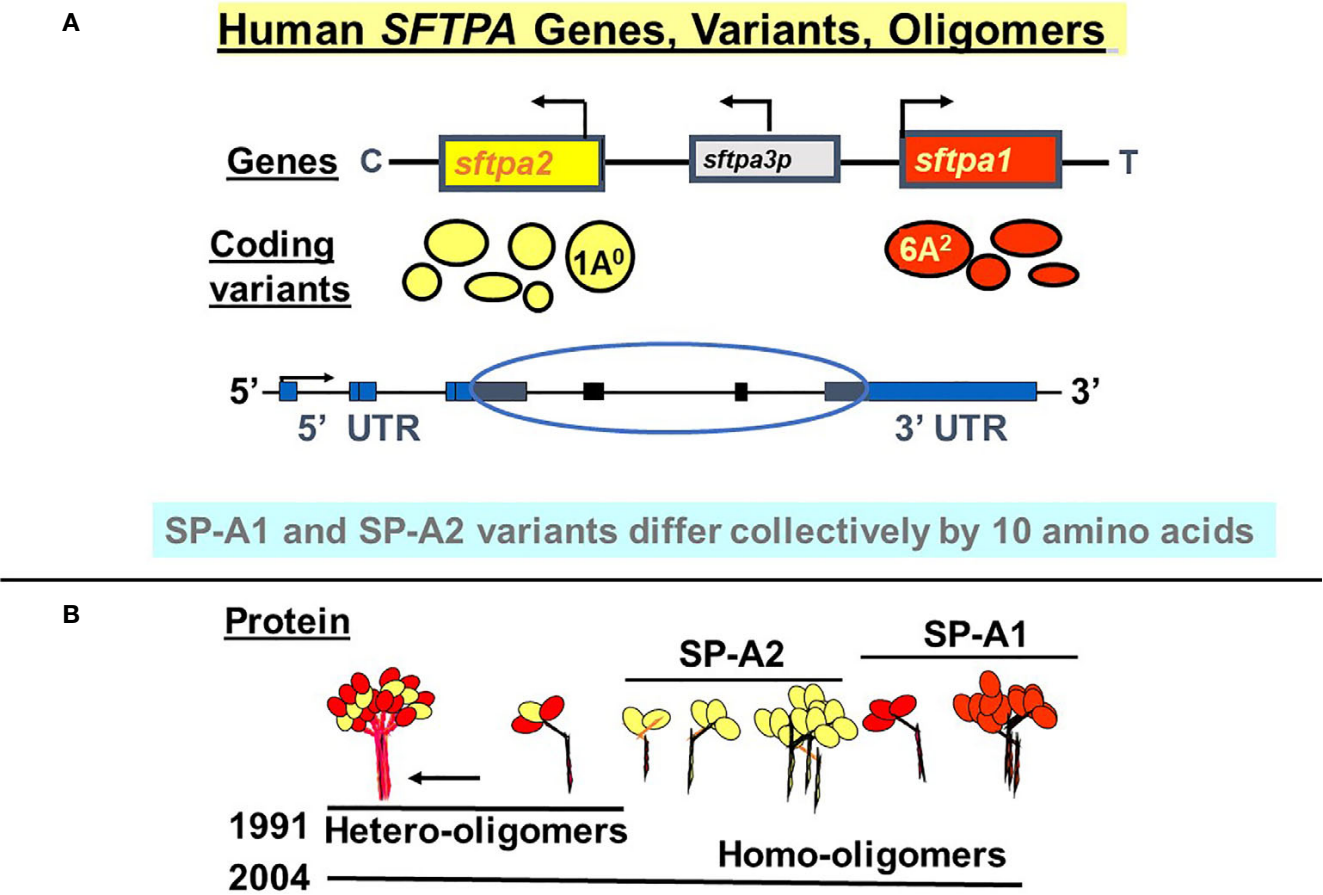
Human *SFTPA* genes, variants, and oligomers. **(A)** depicts the chromosomal *SFTPA* genetic locus from the centromere (C) to telomere (T) as well as the opposite transcriptional orientation (arrow) of the two functional genes (*SFTPA1* and *SFTPA2*). The pseudogene (*SFTPA3P*) is between the two functional genes. The most frequently found coding variants are depicted below each gene. The size of the circle denotes the relative frequency with the 1A^0^ and 6A^2^ being the most frequently observed variants in the general population. A schematic of the gene organization is shown, depicting the 5’ and 3’ UTR (blue boxes) and the coding regions (black boxes, circled). **(B)** depicts hetero-oligomers of SP-A1 and SP-A2 described in 1991 (38) and homo-oligomers described in 2004 (23).

SP-A1 and SP-A2 coding variants have been identified and characterized (5, 29, 35–37). These variants are shown diagrammatically in **Figure 3** and described in detail below. The cartoon in Panel B shows that both hetero-oligomers and homo-oligomers exist. Hetero-oligomers consist of two molecules or monomers of SP-A1 and one molecule of SP-A2 as described initially by Voss et al. (38) and homo-oligomers consist of three molecules of either SP-A1 or SP-A2 (23). SP-A mRNA in adult lung tissue has been localized in the epithelial type II cells by *in situ* hybridization (39), as has the SP-A protein by immunohistochemistry and immunoelectron microscopy (40). Although there have been published works using SP-A from various species (41–48) the focus and the reference points discussed in this review are primarily those of human SP-A1 and SP-A2. Thus, here we focus on functional and structural differences between human SP-A1 and SP-A2 and/or among their corresponding variants, as well as on their differential ability to regulate processes in alveolar cells and impact survival of the organism.

## 2. Structural Differences of SP-A1 and SP-A2 Coding Variants

**Figure 4:**
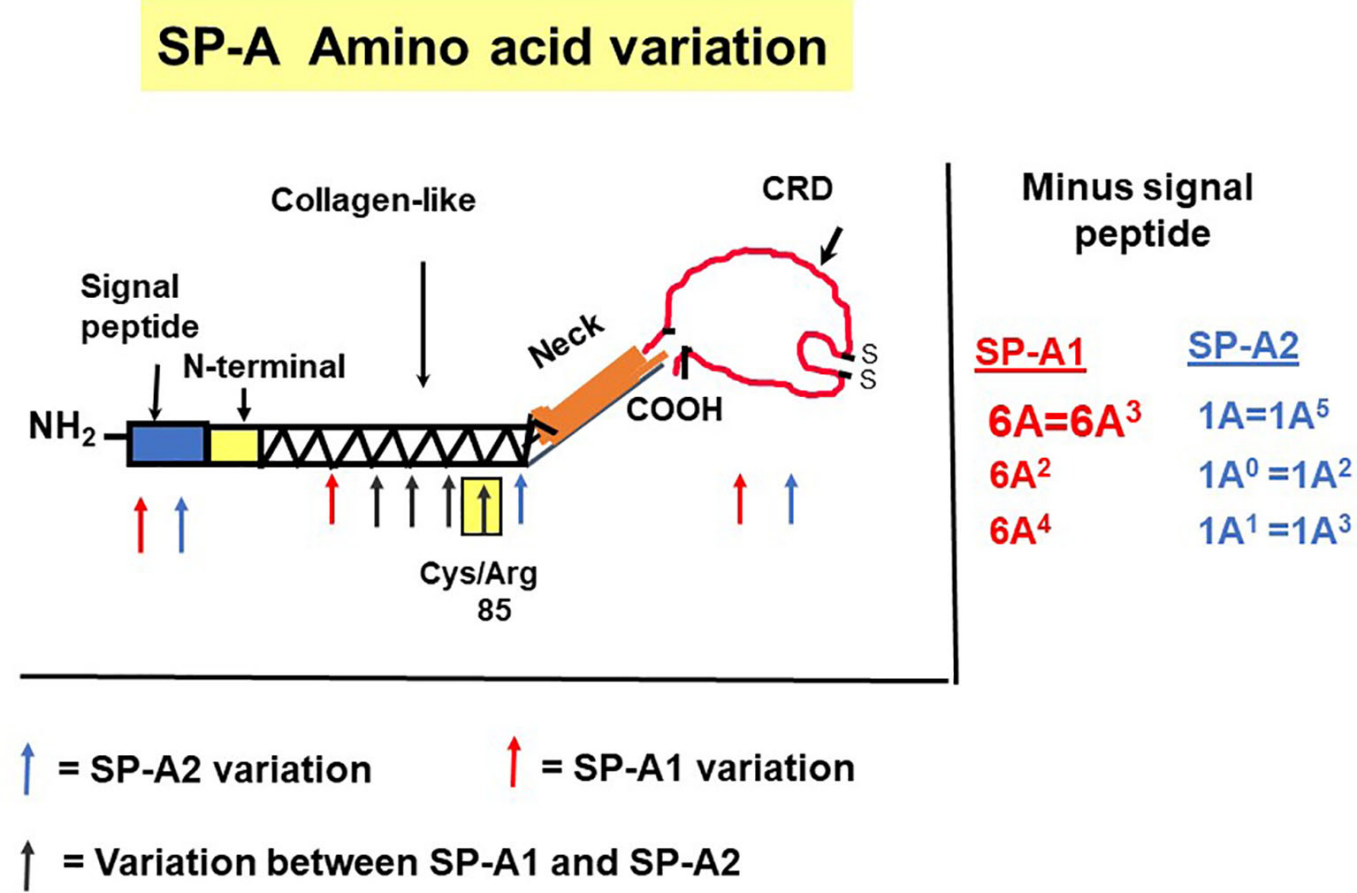
SP-A regions encoding the precursor molecule and the location of the gene-specific and variant-specific amino acid differences is shown (27). The numbering of the amino acids is that of the precursor molecule (i.e., includes the signal peptide). The notation of the most frequently observed variants for each gene is shown on the right. Some of the variants are identical after cleavage of the signal peptide (52).

The precursor molecule of each gene product contains 248 amino acids and each consists of a number of domains including a signal peptide, N-terminal region, collagen-like domain, neck, and C-terminal carbohydrate recognition domain (CRD) (5, 49–51) (**Figure 4**). The signal peptide of 18-20 amino acids (23) and the N-terminal region together consist of 27 amino acids, and the collagen-like domain, neck, and CRD consist of 73, 33, and 115 amino acids, respectively. The gene-specific differences that distinguish the two SP-A genes and their corresponding variants consist only of four amino acids shown in **Table 1**.

**Table 1:** Amino acids differences and their respective codons in parenthesis that distinguish the two human SP-A1 and SP-A2 gene products and their corresponding coding variants.

Amino acid Position	SP-A1 (NP_001158116.1)	SP-A2 (NP_001092138.1)	dbSNP reference number
66	Met (ATG)	Thr (ACA)	rs1059049
73	Asp (GAT)	Asn (AAT)	rs1059052
81	Ile (ATC)	Val (GTC)	rs1059053
85	Cys (TGT)	Arg (CGT)	rs1059054

***SP-A1 and SP-A2 coding variants:*** A number of coding variants have been identified for each SP-A gene product. The most frequently observed (>1%) in the general population are, 6A, 6A^2^, 6A^3^, 6A^4^ for SP-A1 and 1A, 1A^0^, 1A^1^, 1A^2^, 1A^3^, 1A^5^ for SP-A2 (5, 27, 49–51). These variants are found with varied frequency in the general population, with 6A^2^ and 1A^0^ being the most frequently observed (49, 50, 52, 53). The SP-A1 and SP-A2 variants, apart from the gene-specific amino acid differences shown in **Table 1**, differ collectively in six amino acids shown in **Table 2**. Gene-specific and variant-specific nucleotide differences that do not change the encoded amino acid are shown in **Table 3**.

**Table 2:** Amino acid variation among SP-A1 and SP-A2 variants.

†AA#/SNP id	9/rs139899873	19/rs1059047	50/rs1136450	66	73	81	85	91/rs1136452	219/rs4253527	223/rs397728201
SP-A1										
6A	Asn (AAC)	Ala (GCG)	Leu (CTC)	**SP-A1 amino acid gene-specific** **differences** **(** **Table 1** **)**				Pro (CCT)	Arg (CGG)	Gln (CAG)
6A^2^	Asn (AAC)	Val (GTG)	Val (GTC)					Pro (CCT)	Arg (CGG)	Gln (CAG)
6A^3^	Asn (AAC)	Val (GTG)	Leu (CTC)					Pro (CCT)	Arg (CGG)	Gln (CAG)
6A^4^	Asn (AAC)	Val (GTG)	Leu (CTC)					Pro (CCT)	Trp (TGG)	Gln (CAG)
6A^5^	Asn (AAC)	Ala (GCG)	Leu (CTC)					Pro (CCT)	Trp (TGG)	Gln (CAG)
†AA#/SNP id	9/rs1059046	19/rs201847938	50/rs192907309	66	73	81	85	91/rs17886395	219/rs571610539	223/rs1965708
SP-A2										
1A	Thr (ACC)	Ala (GCG)	Val (GTC)	**SP-A2 amino acid gene-specific differences** **(** **Table 1** **)**				Pro (CCT)	Arg (CGG)	Gln (CAG)
1A^0^	Asn (AAC)	Ala (GCG)	Val (GTC)					Aln (GCT)	Arg (CGG)	Gln (CAG)
1A^1^	Thr (ACC)	Ala (GCG)	Val (GTC)					Aln (GCT)	Arg (CGG)	Lys (AAG)
1A^2^	Thr (ACC)	Ala (GCG)	Val (GTC)					Aln (GCT)	Arg (CGG)	Gln (CAG)
1A^3^	Asn (AAC)	Ala (GCG)	Val (GTC)					Aln (GCT)	Arg (CGG)	Lys (AAG)
1A^5^	Thr (ACC)	Ala (GCG)	Val (GTC)					Pro (CCT)	Arg (CGG)	Gln (CAG)

†Amino acid (AA) position is based on the precursor molecule that includes the signal peptide.

Red and Green area indicate gene specific amino acids.

Underlines indicates the variable nucleotide.

**Table 3:** Nucleotide differences between or among SP-A1 and SP-A2 coding variants that do not change the encoded amino acids.

†AA#/SNP id	62/rs1136451	71/rs1059051	94/rs1136454	114/rs1059056		133/rs1059057		140/rs3997777		202/rs1059058		216/rs876657998	
SP-A1													
6A	CCG (Pro)	GGA (Gly)	AGG (Arg)	TTT (Phe)		ACG (Thr)		TCC (Ser)		GAC (Asp)		CCC (Pro)	
6A^2^	CCA (Pro)	GGA (Gly)	AGG (Arg)	TTT (Phe)		ACA (Thr)		TCC (Ser)		GAC (Asp)		CCC (Pro)	
6A^3^	CCA (Pro)	GGA (Gly)	AGG (Arg)	TTT (Phe)		ACA (Thr)		TCC (Ser)		GAC (Asp)		CCC (Pro)	
6A^4^	CCG (Pro)	GGA (Gly)	AGG (Arg)	TTT (Phe)		ACA (Thr)		TCC (Ser)		GAC (Asp)		CCC (Pro)	
6A^5^	CCG (Pro)	GGA (Gly)	AGG (Arg)	TTT (Phe)		ACA (Thr)		TCC (Ser)		GAC (Asp)		CCC (Pro)	
†AA#/SNP id	62/rs2434114	71/rs143780551	94/rs17886221		114/rs147679203		133/rs763971475		140/rs1965707		202/rs17880902		216/rs17096771
SP-A2													
1A	CCG (Pro)	GGG (Gly)	AGA (Arg)		TTC (Phe)		ACA (Thr)		TCC (Ser)		GAT (Asp)		CCT (Pro)
1A^0^	CCG (Pro)	GGG (Gly)	AGA (Arg)		TTC (Phe)		ACA (Thr)		TCC (Ser)		GAT (Asp)		CCT (Pro)
1A^1^	CCG (Pro)	GGG (Gly)	AGA (Arg)		TTC (Phe)		ACA (Thr)		TCT (Ser)		GAT (Asp)		CCT (Pro)
1A^2^	CCG (Pro)	GGG (Gly)	AGA (Arg)		TTC (Phe)		ACA (Thr)		TCC (Ser)		GAT (Asp)		CCT (Pro)
1A^3^	CCG (Pro)	GGG (Gly)	AGA (Arg)		TTC (Phe)		ACA (Thr)		TCT (Ser)		GAT (Asp)		CCT (Pro)
1A^5^	CCG (Pro)	GGG (Gly)	AGA (Arg)		TTC (Phe)		ACA (Thr)		TCT (Ser)		GAT (Asp)		CCT (Pro)

†Amino acid (AA) position is based on the precursor molecule that includes the signal peptide.

The nucleotide differences in the columns highlighted in yellow are gene-specific.

Underlines indicates the variable nucleotide.

All the gene-specific differences are found in the collagen-like domain of the proteins (49) (**Figure 4**), which consists of 23 Gly-X-Y triplets with an irregularity (Pro-Cys-Pro-Pro instead of Gly-X-Y) present between triplets 13 and 14. This irregularity generates a kink in the structure (49, 54). The four gene-specific amino acid differences at positions 66, 73, 81, 85 (**Table 1**) are located within the collagen-like domain at Gly-X-Y triplets, 13, 14, 17, and 18, respectively. Of the gene-specific amino acid differences, amino acid 85 is shown to affect both structure and function of the SP-A protein. When mammalian (CHO) cell-expressed wild type (WT) and mutated proteins containing interchanged residues in position 85 (SP-A1^C85R^ and SP-A2^R85C^) were compared, the oligomerization patterns were largely determined by the amino acid at position 85 (55). These results were consistent with, and confirmed oligomerization patterns observed, in previous studies (23). In humanized transgenic mouse models where each mouse carries and expresses a different SP-A1 or SP-A2 variant (56), SP-A1 forms dimers, hexamers, and larger oligomer complexes under non-reducing conditions, while SP-A2 forms mostly dimers, trimers, tetramers, and hexamers (56). Differences in oligomerization were also observed in SP-A1 and SP-A2 proteins expressed in insect cells (57), although the pattern differed from that in mammalian cell-expressed SP-As. SP-A undergoes significant co- and post-translational modifications, some of which cannot be performed by insect cells. For example, insect-cell expressed SP-A is deficient in hydroxyproline (58) and hydroxylation of prolines retards the electrophoretic mobility of SP-A (59) as well as increases the melting temperature (Tm) compared to molecules lacking proline hydroxylation (60). Insect cell-expressed SP-A2 demonstrated higher rough LPS aggregation activity than SP-A1 (54, 61). SP-A2 also exhibited a higher activity to self-aggregate and induce aggregation of phospholipids and bacterial lipopolysaccharides than co-expressed or co-ex (SP-A containing both SP-A1 and SP-A2) or SP-A1 proteins (SP-A2 > SP-A1/SP-A2 > SP-A1). This indicates that SP-A1, either in homo-oligomers (i.e., by itself) or hetero-oligomers (i.e., in the presence of SP-A2), exhibits a lower ability to self-aggregate (54). Moreover, SP-A2 was shown, *via* circular dichroism and fluorescence studies, to be more stable than SP-A1 (54). Based on substitution experiments of amino acids X and Y in Gly-X-Y triplets in other systems (62, 63), it was postulated that the presence of Cys85 (in SP-A1) within the collagen-like domain creates a micro instability contributing, perhaps, to a lower SP-A1 stability compared to SP-A2 (Arg85) (54).

*In vitro* experiments of mammalian expressed proteins showed that the order of the proteins with regards to their activity in aggregating rough LPS was SP-A2^WT^>SP-A1^WT^>SP-A1^C85R^>SP-A2^R85C^ (55). Substitutions in residue 85 affected LPS aggregation as well as phagocytosis of *P. aeruginosa* by male rat alveolar macrophages (AM). The phagocytic index of mutated and wild type (WT) SP-A1 and SP-A2 was SP-A1^C85R^ > SP-A2^WT^ > SP-A1^WT^ = SP-A2^R85C^ (55). Cysteine 85 of SP-A1 allows formation of additional intermolecular disulphide bridges that affects oligomerization patterns (23). Furthermore, introduction of cysteine 85 in SP-A2 changes its oligomerization pattern to one that is identical to SP-A1, while introduction of arginine 85 in SP-A1 increased the efficiency of rat AMs to phagocytose bacteria (55). Importantly, the natural presence of arginine 85 in SP-A2 introduces an additional trypsin digestion site, which makes the protein more susceptible to degradation by trypsin at 37°C (61). These results collectively highlight the importance of residue 85. In addition to the importance of residue 85, the presence of Trp (instead of Arg) in position 219 of the SP-A1 6A^2^ variant, which is associated with increased risk for idiopathic pulmonary fibrosis, has been shown to mediate differences in protein self-aggregation before and after ozone exposure (64).

## 3. Surfactant Structure- and Function-Related Differences in the Presence of SP-A1 and SP-A2

**Figure 5:**
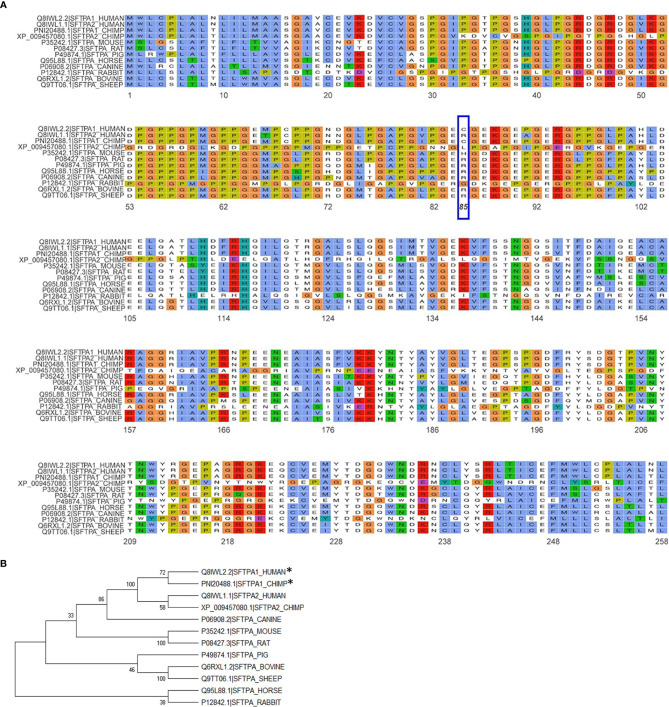
Alignment and phylogeny of SP-A1 and SP-A2 sequences. **(A)** shows the alignment of the NCBI available SP-A sequences for the species shown using the ggmsa R package (http://yulab-smu.top/ggmsa/index.html). Per NCBI statement “this record is predicted by automated computational analysis. This record is derived from a genomic sequence (NC_036889.1) annotated using gene prediction method: Gnomon, supported by mRNA and EST evidence”. Gnomon, a gene prediction tool, looks for ORFs, splice sites etc. The amino acid of residue 85 is highlighted by the blue box. The color scheme follows the default colors used in alignments in Clustal X. **(B)** shows the phylogenetic tree of the sequences shown in **(A)**. The tree was created with the MEGA X software (www.megasoftware.com), using the neighbor-joining method. The numbers next to the branches represent the percentage of replicate trees in which the associated taxa clustered together in the bootstrap test (1000 replicates). The asterisk mark the two sequences, human SP-A1 and primate SP-A1 sequence, that have a C (Cys) at residue 85. All other species sequences have an R (Arg) except for the mouse that has a H (His) and the primate *SFTPA2* gene that has an L (Leu), as shown in **(A)**.

The importance of the SP-A collagen-like domain in surfactant-related function/structure was highlighted in transgenic mice carrying a truncated form of SP-A with a deletion of amino acids 6-80 on the SP-A^-/-^ (knockout) background (65). These mice could not form tubular myelin, an extracellular structural form of surfactant, which is considered to be a surfactant reservoir. The mouse SP-A has a histidine in position 85 and the rat, an arginine. Both of these residues are basic polar and thus similar to SP-A2, which contains an arginine at position 85. The SP-A1, on the other hand, has a cysteine at position 85 and this could mediate intra- and inter-molecular disulfide bonds that none of the amino acids at position 85 in rodent SP-A or SP-A2 could form (23). An amino acid alignment of all SP-A species sequences available along with their sequence similarity is depicted in **Figure 5A**. Given the importance and magnitude of the impact of residue 85 on structure and function (based on experimental evidence), a few comments are warranted about this residue in the various species. Humans and primates have two *SFTPA* genes whereas all other mammalian species known to date have a single *SFTPA* gene. The *SFTPA1* gene in humans and primates (chimpanzee) that encodes SP-A1 has a Cys at residue 85 but none of the other sequences has a Cys at this residue. The second *SFTPA* gene in humans encodes SP-A2 and has an Arg at residue 85. Similarly, all of the other sequences have an Arg except for the mouse sequence that has a histidine and the primate *SFTPA2* that has a Leu, making all these sequences, based on residue 85, similar to SP-A2. Panel B of **Figure 5** depicts a phylogenetic tree of the species shown in the alignment in Panel A of **Figure 5**. Amino acid differences in other residues have not been functionally examined *via* amino acid substitution experiments as residue 85 has. However, although these amino acids may contribute to various SP-A-mediated functions these may not impart the magnitude of functional differences caused by residue 85 (55). Interestingly, a recombinant trimeric fragment of human SP-A1 that contains the CRD, the neck, and the last 8 Gly-X-Y repeats (including Cys85) is efficacious at neutralizing respiratory syncytial virus and preventing infection of differentiated human bronchial epithelial cells (66).

Tubular myelin, an extracellular structural form of surfactant, was not observed in hTG mice expressing either SP-A1 or SP-A2, although both types of mice had lamellar bodies, and SP-A1-expressing mice had larger lamellar bodies (56). Tubular myelin was, however, present in SP-A1/SP-A2 co-expressing transgenic mice, indicating the need for both gene products for tubular myelin formation (56). Moreover, a rescue study of SP-A knockout (KO) mice with exogenous human SP-A from bronchoalveolar lavage (BAL) showed that tubular myelin was restored, when the exogenously administered SP-A contained both SP-A1 and SP-A2. But this was not the case if the exogenous SP-A contained primarily SP-A1 or SP-A2 (56) further supporting the observation that both SP-A1 and SP-A2 gene products are necessary for tubular myelin formation. Furthermore, SP-A has been localized in tubular myelin using a number of methods (40, 67).

**Figure 6:**
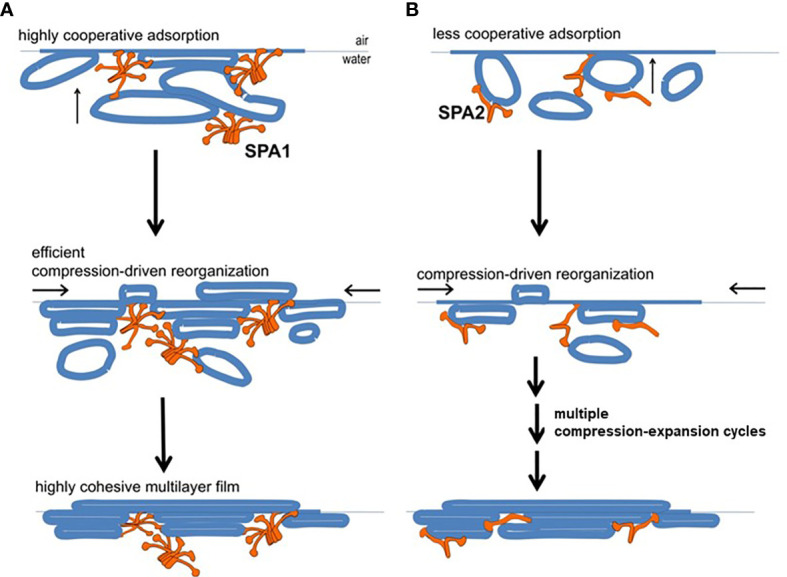
Model of behavior of surfactant lipid complexes in the presence of SP-A1 or SP-A2 proteins. **(A)** depicts the multivalent binding capacities of SP-A1. The higher SP-A1 multivalent binding capacity is advantageous as it brings together several membrane aggregates and forms complex films. This is due to its higher oligomerization state that is mediated in part by the presence of Cys85. These complex films, upon compression-driven reorganization, could rapidly generate highly cohesive multilayer films that can reach very low surface tension. **(B)** depicts how SP-A2, which is less oligomerized and exhibits lower binding capacity, forms surface films with lower density of membranes (i.e., less complex films). These simpler films can still be reorganized in a cohesive multilayer film to impart low surface tension but they require repetitive compression-expansion cycling. Therefore, part of the energy needed for the initial compression-expansion cycles will be spent to promote folds and membrane-membrane contacts needed prior to generating the cohesive multilayer film, making this process less efficient (22).

A study using a captive bubble surfactometer to investigate the biophysical properties of lung surfactant from humanized transgenic mice expressing either protein or both SP-A proteins showed that the presence of SP-A1 minimizes hysteresis (22). Hysteresis has been described and studied extensively elsewhere by experts in the field of pulmonary mechanics and in biophysical studies of pulmonary surfactant (68–71). In the study by Lopez-Rodriquez (22) SP-A1 proved to be better in enabling lipid membrane reorganization by more efficiently bringing together membrane aggregates to form complex films **(** **Figure 6** **)** and showed a higher efficiency in phospholipid adsorption at the air-liquid interface. Moreover, when experiments were performed at surfactant concentrations to detect differences between SP-A1 and SP-A2, SP-A1-containing preparations lowered surface tension more efficiently, even in the presence of serum (22), indicating that SP-A1 has a better ability to prevent inhibition of surfactant function by serum proteins.

Moreover, SP-A2 is more effective than SP-A1 in promoting dipalmitoyl phosphatidylcholine (DPPC) and dipalmitoyl phosphatidylglycerol (DPPG) lipid vesicle aggregation (54, 61). DPPC monolayers containing porcine SP-B, in the presence of SP-A2, are morphologically closer to the ones containing native SP-A (i.e., SP-A from BAL). This is not the case with SP-A1, where although SP-A1 allows the formation of SP-B/DPPC monolayers, these differ compared to those with native SP-A (72). However, the lipid SP-B containing films in the presence of both SP-A1 and SP-A2 (i.e., co-ex proteins) showed combined characteristics of films containing either SP-A1 or SP-A2. It is possible that varying levels of SP-A1 and/or SP-A2 (73) in BAL may differentially affect the physiological properties of surfactant and be critical for lung health.

## 4. SP-A1 and SP-A2 Differentially Affect the Activity of Alveolar Macrophages (AMs) *Ex-Vivo*

The sentinel innate immune cell in the alveolus is the AM, and SP-A is shown to interact with the AM (74–77). SP-A1 and SP-A2 variants differentially affect several of the processes and functions of AM, as discussed below. SP-A2 enhances bacteria phagocytosis by AM more than SP-A1. Insect-cell expressed SP-A2 was more active than SP-A1 in its ability to increase association of rat AM with *Pseudomonas aeruginosa* in all concentrations studied (78). Although the observations from CHO-cell expressed SP-A1 and SP-A2 proteins (79) with regards to bacterial phagocytosis were similar to those observed by insect-cell expressed SP-As, the concentration of the CHO-cell expressed protein needed for this effect was orders of magnitude lower (79). SP-A undergoes a significant number of co- and post-translational modifications (59, 80–84), but as mentioned earlier, the insect-cell derived SP-A proteins lack certain post-translational modifications (58). The significantly higher concentration of insect-expressed protein needed for a similar functional activity as that observed with CHO-derived SP-A protein (79) indicates that the co- and post-translational modifications of SP-A are necessary for optimal function.

As discussed above, a number of SP-A1 and SP-A2 variants have been identified and the variants of each protein studied differentially impact AM function and thus are of importance. Of the SP-A2 variants studied, in terms of their ability to enhance bacterial phagocytosis, the 1A^1^ variant was more efficient than 1A^0^, and of the SP-A1 variants, the 6A^2^ was more efficient than 6A^4^. Apart from the SP-A1 and SP-A2 gene-specific differences shown in **Table 1**, the SP-A2 variants 1A^1^ and 1A^0^ differ in three residues, at positions 9, 140, and 223 (**Tables 2** and **3**), while the SP-A1 variants differ in three residues, at positions 50, 62, and 219 (**Tables 2** and **3**) (27, 49, 53). While the degree to which these amino acids contribute to various functions is not entirely known, the amino acid differences of these SP-A1 and SP-A2 variants collectively are located within the collagen-like domain (amino acid 50) and the CRD (amino acids 219 & 223) of the mature protein. Nucleotide polymorphisms at residues 62 and 140 do not change the encoded amino acid and residue 9 is part of the signal peptide (27). However, the gene-specific difference Cys/Arg at residue 85 in SP-A1 and SP-A2, respectively, has a significant effect on AM function including on bacterial phagocytosis (55), perhaps partly due to the structural changes this residue imparts on SP-A1 and SP-A2 (i.e., oligomerization, stability, other). As noted, the contribution of amino acids other than Arg/Cys85 in functional and/or structural differences has not been studied in detail. We speculate that although the magnitude of change of Arg/Cys85 on function/structure is significant, the amino acid differences in other SP-A1 and SP-A2 residues serve as a rheostat to modulate the overall function of each SP-A1 or SP-A2 variant. This postulate is consistent with findings in several functional read-outs, including bacterial phagocytosis discussed above and others discussed below. As a group, SP-A2 variants exhibited higher activity than SP-A1 variants in their enhancement of the bacterial phagocytic index (78, 79, 85), but the variants within each group, expressed either in mammalian CHO cells (79) or insect cells (78), also showed differences in their effects on the phagocytic index. Whether the higher SP-A2 activity is due, in part, to its CRD being able to bind a wider variety of sugars (on pathogen surfaces) compared to SP-A1 (86) remains to be determined. Comparable observations were made on SP-A1 and SP-A2 variants in their ability to enhance pro-inflammatory cytokine production by a macrophage-like cell line (57, 87, 88).

Exposure of SP-A to ozone (O_3_) (89) decreases its ability to interact with alveolar macrophages (90, 91). Following oxidation of CHO-derived SP-A, the ability of SP-A2 to enhance phagocytosis of *P. aeruginosa* by rat alveolar macrophages remained higher than SP-A1, albeit, compared to SP-A1, the drop in activity before and after O_3_ exposure was larger in SP-A2, indicating that SP-A2 is more sensitive to O_3_ exposure than SP-A1 (85). Similarly, ozone exposure of SP-A variants had a negative impact on SP-A-regulated cytokine production (57, 88).

***Other:*** Despite their differential impact on macrophage activity, the two SP-A proteins did not seem to differ in their activity against the influenza A virus (92). This, however, may need to be studied further because the SP-A proteins studied were expressed in CHO cells. These cells lack α(2,6)-sialyltransferase (93, 94) and thus they cannot synthesize α(2,6)-linked sialic acids in the expressed proteins, but they do form α(2,3)-linked sialic acids (92). Insect-cell expressed SP-As, on the other hand, that lacked sialic linkages altogether failed to inhibit the hemagglutination activity of influenza A virus (92). The presence of the specific sialic acid linkages for appropriate SP-A function may be critical in response to influenza A virus. These linkages or other co-/post-translational modifications or genetic variations may in fact differentially contribute to SP-A1 and SP-A2 variant function in response to influenza A virus infection as suggested by human association studies (95). Furthermore, an SP-A2 223Q variant (glutamine residue at position 223) was shown in mice to provide significant protection from ozone-induced airway hyper-responsiveness compared to another SP-A2 223K variant (lysine at 223) and the SP-A KO mice (96).

## 5. Differences in the Bronchoalveolar Lavage (BAL) and/or Alveolar Macrophage (AM) Proteome of Various SP-A Mouse Models and Conditions

### 5a. SP-A Knockout (KO) and Wild Type (WT) Mice

**BAL proteome:** The mouse BAL proteome is affected by the presence of SP-A. The proteins mostly affected at basal conditions in the KO *vs* WT are involved in host defense, redox equilibrium, and protein modifications (97). Proteins with a role in host defense against *Klebsiella pneumonia*e (98, 99), were elevated in the SP-A KO mouse under basal conditions, indicating potential compensatory mechanisms for host defense deficits in the KO. However, these do not seem to be adequate in the long term, because SP-A-KO mice infected with *K. pneumoniae* are shown to exhibit significantly reduced survival compared to WT mice (100). Moreover, the levels of these proteins (lysozyme and β2-microglobulin) were decreased in infected KO and the levels of other proteins involved in host defense and redox balance were altered in infected KO compare to WT infected mice. These together indicate that in the absence of SP-A there may be a loss of control to effectively regulate expression of such proteins in response to infection. Furthermore, lipopolysaccharide (LPS) treatment of KO mice negatively impacts markers of inflammation long after treatment and the KO become less capable of responding to a second insult (101).

Similar biological pathways were affected in the O_3_-challenged KO mouse model. The response pattern of the BAL proteome of KO mice exposed to O_3_ *vs* filtered air-exposed, is magnified compared to that of similarly treated WT mice (102). An interesting observation from the same work was the similarities in the levels of changed proteins observed between filtered air-exposed KO mice and O_3_-exposed WT mice, indicating that the absence of SP-A may place the alveolar cells in a state of chronic oxidative stress, as may occur after O_3_ exposure in WT mice. Moreover, the magnitude of several protein changes in response to infection or O_3_ exposure in the KO and WT differed, with the KO exhibiting a higher degree of response. A possible explanation would be that SP-A plays a role in processes involved in infection and/or oxidative stress and its absence generates a deficit in host defense functions and/or loss of regulatory control leading to an overexuberant response in KO in the face of insult. A recent study, using optical redox imaging showed that AM from KO are more oxidized after O_3_ exposure compared to AM that were chronically or constitutively exposed to SP-A2 (103).

**AM proteome:** Because SP-A has been shown to modulate several AM functions, it was postulated that several of the protein changes found in the KO and WT BAL proteome are partly due to changes in the AM proteome. A rescue proteomics experiment where SP-A KO mice were treated with a single dose of human SP-A, revealed that the proteome of rescued AM, resembled that of wild type (WT) AMs (104, 105). The fact that a single exogenous dose of SP-A is sufficient to nearly restore the male AM proteome to its WT state (104, 105) provides support for a role of SP-A in the regulation of the AM proteome and consequently the BAL proteome. More importantly, this highlights the potential of SP-A therapy. Sex differences were also observed in this rescue study (104, 105). A number of proteins, such as major vault protein, chaperonin subunit (beta) (CCT2), and Rho GDPα dissociation inhibitor, found to be increased in females, are shown to interact with the estrogen receptor (106–108) indicating that hormones may play a role in mediating SP-A action. Consistent with this notion is the finding that: a) gonadectomy eliminated (females) or significantly reduced (males) survival differences of infected mice in the presence or absence of O_3_-exposure; and b) sex hormone replacement in gonadectomized mice had a significant impact on the survival of the male and female mice after *K. pneumoniae* infection (109). The patterns of survival after hormone replacement of the gonadectomized mice resembled those of intact mice (109). In summary, SP-A appears to regulate hormone-dependent genes/processes. Although the mechanisms involved are not understood yet, this is an important topic and warrants further investigation.

**Figure 7:**
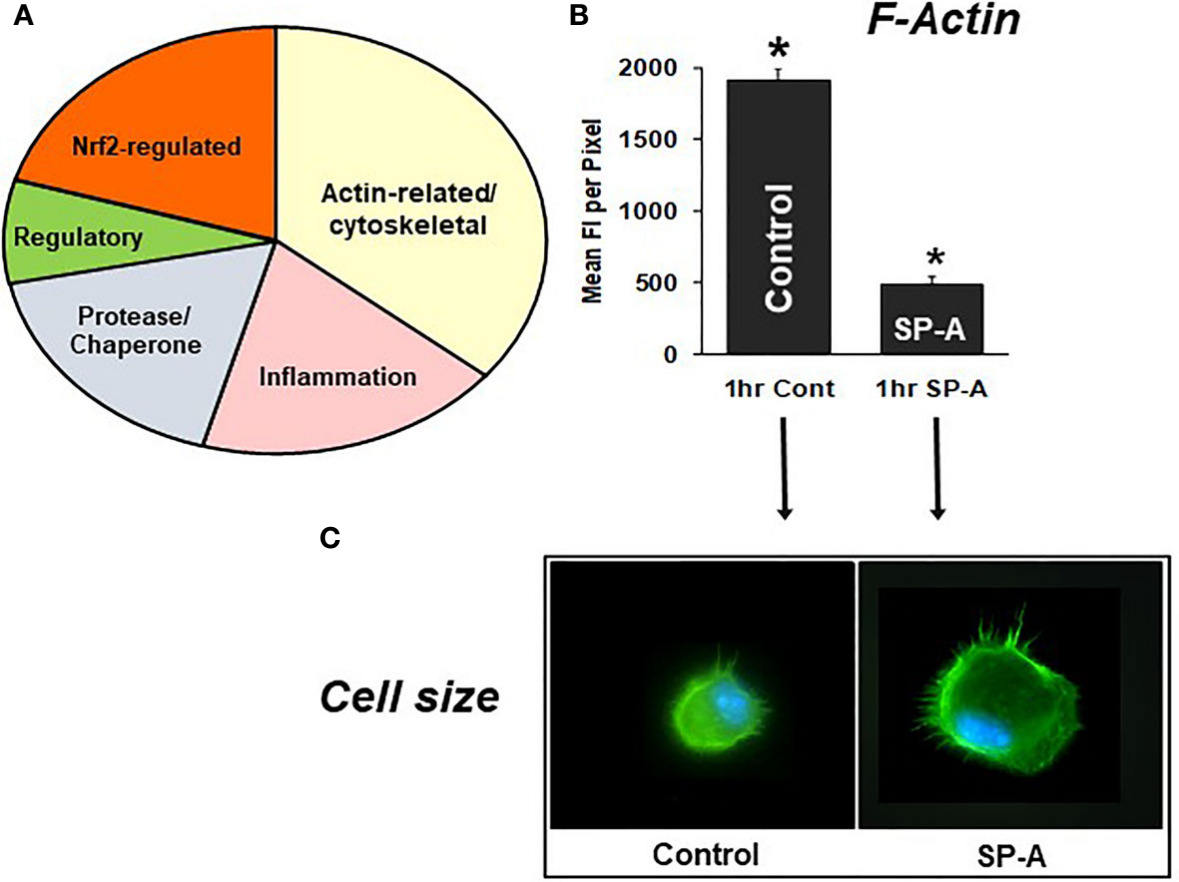
SP-A rescue of the SP-A KO AM. **(A)** depicts the groups of proteins that changed in response to SP-A. **(B)** depicts the mean fluorescence per pixel of F-actin (cells stained with Alexa Fluor 488 phalloidin) after 1hr of treatment with SP-A (1hr SP-A) or untreated (1hr control) SP-A KO AM (105). **(C)** depicts the actual fluorescence in the control and SP-A-treated AM as well as the size change after the treatment (110). AMs were visualized using a Nikon TE-2000 PFS fluorescent microscope with a 60X (1.4 NA) phase contrast lens with a 1.5× tube lens in place and the images captured using a Photometrics Coolsnap HQ2 digital camera (0.07 μm/pixel) and saved as TIFF files (105). *p < 0.0001.

Differences in specific protein levels were observed in AM in the presence or absence of SP-A. These proteins were involved in several pathways including pathways pertaining to actin cytoskeleton signaling, Nrf2-mediated oxidative stress, regulation of inflammation, protease/chaperone function, cell differentiation and other **(** **Figure 7A** **)** (105). A single dose of SP-A treatment of AM in culture obtained from SP-A KO mice, significantly decreased F-actin intensity/pixel as early as one-hour post treatment **(** **Figure 7B** **)** and increased AM cell size **(** **Figure 7C** **)** (105, 110), indicating that in response to SP-A a number of cytoskeletal events may occur. These may include a redistribution of cortical actin over a larger cell area and/or altered gene expression of actin-related proteins (105). These changes may underlie functional activities of the AM, such as bacterial phagocytosis, observed at one hour after AM are incubated with SP-A and bacteria (78, 79, 85).

### 5b. Humanized Transgenic (hTG) Mice Where Each Carries and Expresses a Single SP-A1 or SP-A2 Variant, or Both

***BAL proteome:*** The BAL proteomes of SP-A1 and SP-A2 hTG mice are differentially affected by human SP-A variants (111). In terms of the number of proteins that changed (more than 25% in BAL), the male SP-A2 mice exhibited a more robust response (SP-A2 > SP-A1 = KO) following *K. pneumoniae* infection in the presence of O_3_ exposure (111). Moreover, the majority of the changed proteins showed increases in males and decreases in females. However, in the absence of O_3_ exposure following infection the SP-A1 and SP-A2 variants differentially affected the BAL proteome in males (SP-A2 > SP-A1 > KO), but less so in females (SP-A2 = SP-A1 > KO) (111). The top pathways implicated in these differences are the acute phase response (APR) that includes the NF-kB signaling pathway, and the Nrf2-mediated oxidative response, among others. A time-dependent Nrf2 translocation from the cytoplasm to the nucleus was observed after O_3_ exposure (111). Nrf2-regulated proteins were also identified in the proteome of the AM in an SP-A rescue experiment of SP-A KO mice (104), further supporting a role of SP-A in this signaling pathway. Compared to KO, the effect of SP-A2 on signaling pathways, such as APR and Nrf2, exhibited sex differences and in males appeared to be independent of O_3_-exposure, whereas this was not entirely the case in SP-A2 females, especially for Nrf2. Under similar conditions SP-A1 exhibited sex differences both in response to O_3_ and filtered air exposures. The SP-A1 effect on Nrf2 was eliminated after O_3_ exposure in both males and females. These findings indicate a higher ability of SP-A2 to elicit innate immune responses, with sex differences being observed with SP-A1 and SP-A2.

***AM Proteome:*** The proteome profile of AMs from humanized transgenic mice that express human SP-A1 or SP-A2 showed dose and sex-dependent differences (112). The SP-A2 (but not the SP-A1) profile shared similarities with that of WT, especially in proteins related to actin cytoskeleton and Nrf2-regulated proteins. However, the expression pattern of AM-derived from hTG mice expressing high levels of SP-A1 or SP-A2 was the inverse for many proteins, i.e., proteins with high levels in one type of hTG mouse (i.e., SP-A1 or SP-A2) were found with low levels in the other and vice versa. This is in contrast to the pattern observed in AM derived from hTG mice expressing low levels of SP-A1 or SP-A2 (112) where the response pattern of the two proteomes was similar. One possibility is that low levels of SP-A1 or SP-A2 affect the AM *via* a high affinity non-SP-A variant-dependent receptor, whereas at high levels, SP-A1 or SP-A2 may act *via* a different receptor or *via* the same one, but with different binding capacities resulting in response differences. Consistent with this, SP-A2 is shown to exhibit a higher binding capacity to AM compared to SP-A1. Moreover, SP-A1, in contrast to SP-A2, showed no binding to AMs that were not previously exposed to SP-A (i.e., in KO AMs) (113). Collectively, these indicate potential complexities of SP-A interactions with AMs. SP-A1 may have an inhibitory effect on AM binding by SP-A2 since the SP-A2 binding capacity was reduced when AMs were exposed continually to SP-A1 prior to the assay (113).

**Figure 8:**
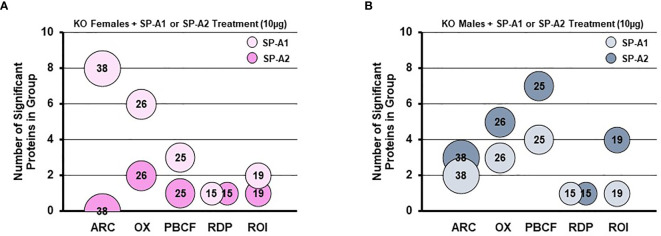
Sex differences of the AM proteome in response to *in vivo* rescue of SP-A KO mice with different human SP-A variants. **(A, B)** show differential effects of SP-A1 and SP-A2 in females **(A)** and males **(B)** in terms of various functional protein groups. Bubble size and number depict the number of proteins within each group (114). ARC, actin-related cytoskeletal; OX, oxidative stress; PBCF, protease balance/chaperone function; RDP, regulatory/developmental processes; ROI, regulation of inflammation.

A rescue proteomic experiment of SP-A KO mice with a single dose of either SP-A1 or SP-A2 showed variant-specific protein changes as well as sex-specific changes for various groups of proteins (114). The differentially expressed proteins were grouped in one of the following categories: protease balance/chaperone function group, actin-related cytoskeleton, Nrf2-related proteins, regulatory/differentiation-related proteins, and regulation of inflammation-related proteins (114). Responses to the different SP-A gene products of these groups of proteins were sex-specific. Females were more responsive to SP-A1 with respect to proteins involved in actin metabolism and oxidative stress, and males were more responsive to SP-A2 treatment, particularly with regards to the regulation of proteins involved in protease balance/chaperone function and in inflammation **(** **Figure 8** **).** The functional group that was similar between males and females exposed either to SP-A1 or SP-A2 was the one containing regulatory/developmental-related proteins (114). Of interest, the response pattern of most of the proteins involved in “the regulation of actin-based motility by Rho” pathway in male and female AM rescued by SP-A1, was the inverse of one another (114).

The BAL and AM proteomics data collectively indicate that: a) SP-A plays an important role in the expression of proteins found in bronchoalveolar lavage (BAL) and those of the AM, under baseline conditions and in response to various insults, such as infection and oxidative stress. b) Sex-specific and SP-A variant-specific changes exist. c) An SP-A dose effect exists, where the proteome profile of AM from mice expressing high levels SP-A1 and SP-A2 differed, whereas the AM proteome from mice expressing low levels showed no major differences. d) Several pathways were identified in response to SP-A and between SP-A1 and SP-A2 variants under various conditions that included the actin cytoskeleton, Nrf2, NF-kB, and other. This complex interplay of SP-A variants and their total protein levels, sex, and exposure (whether environmental or infectious) that together lead to varying outcomes under the studied conditions, point to opportunities for further research into underlying mechanisms. Understanding mechanisms of SP-A variant-mediated regulation of sex-dependent molecules/processes may help in personalized medicine decisions when the use of SP-A therapy may be considered.

## 6. Differential Impact of SP-A1 and SP-A2 on Cytoskeleton, miRNome, Gene Expression and Toponome of Alveolar Cells

### 6a. Actin Cytoskeleton

**Figure 9:**
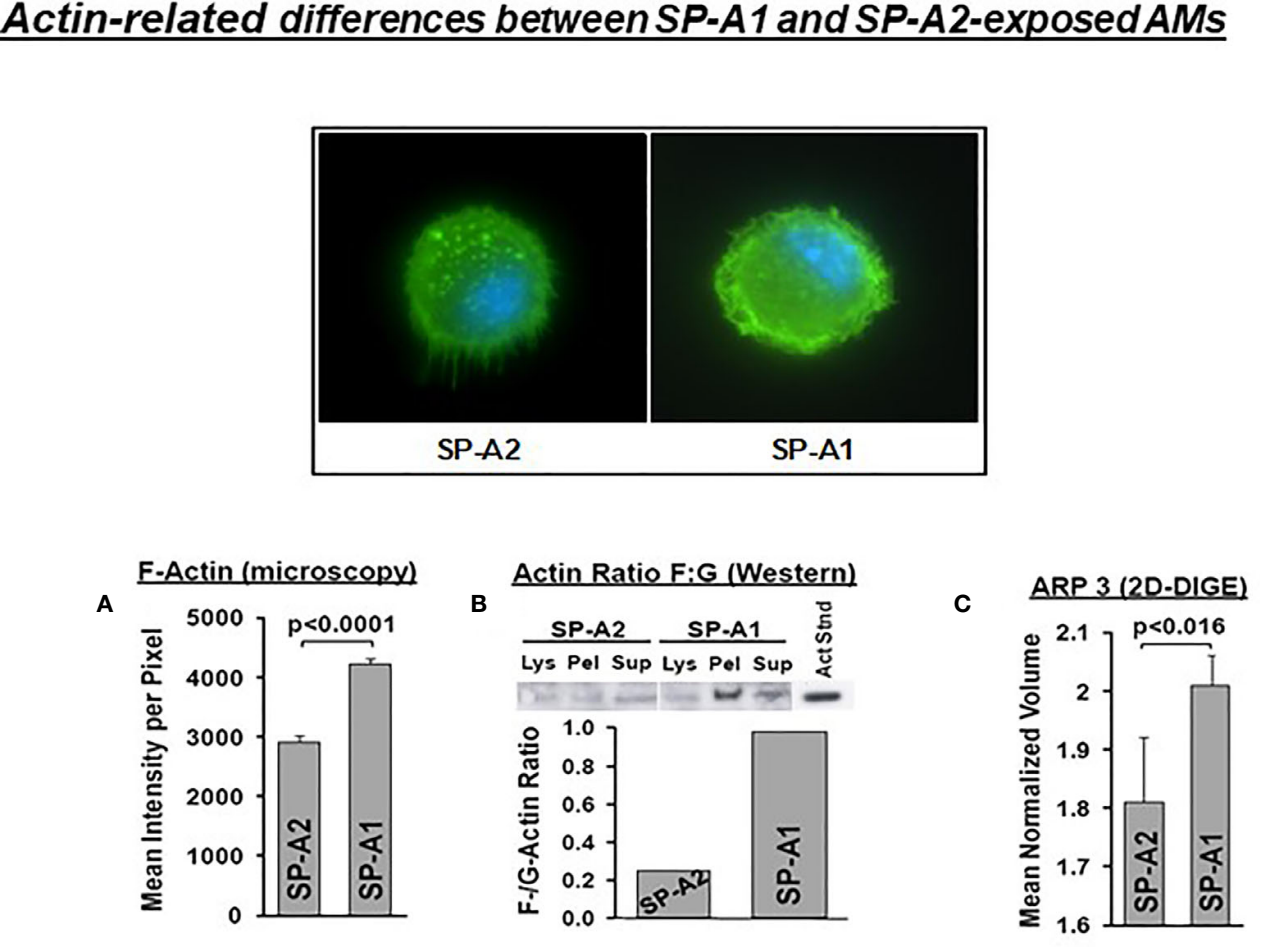
Actin-related differences between SP-A1 and SP-A2-exposed AM (110). The pattern of fluorescence (cells stained with AlexaFluor 488 phalloidin) of AM exposed chronically to SP-A2 or SP-A1 is shown and it is not identical. **(A)** depicts the mean intensity of fluorescence per pixel and shows significant differences between SP-A2 and SP-A1 AM. **(B)** shows the ratio (densitometric measurements) of F-actin to G-actin in different cell fractions from a Western blot stained with an anti-actin N-terminal antibody (Sigma #2103). This ratio is lower in SP-A2 AM. **(C)** depicts significant differences between SP-A1 and SP-A2 in the levels of ARP3 obtained from proteomics data. Arp3 regulates actin polymerization and is part of the Arp2/3 complex, where several signaling pathways leading to actin polymerization converge.

SP-A has been shown to induce changes in the actin cytoskeleton of AMs (115). AMs from SP-A1 (6A^2^) and SP-A2 (1A^0^) hTG mice showed *via* proteomic analysis, among others, significant differences in heat maps of actin-related/cytoskeletal proteins (112), pointing to differences in actin cytoskeletal processes. **Figure 9** shows that the pattern of F-actin fluorescence distribution differs between SP-A1 and SP-A2 AM stained with AlexaFluor phalloidin and that F-actin is decreased in SP-A2 compared to SP-A1 AM, as assessed by measurements of the fluorescence intensity/pixel (Panel A) or by Western blot (Panel B) (110). Moreover, the ARP3 that regulates actin polymerization is also decreased in SP-A2 AM (**Figure 9C**), consistent with the lower F-actin levels (110). We postulate that the state of the cytoskeleton in SP-A2 AM makes the AM more agile for movement and function (i.e., bacterial phagocytosis).

**Figure 10:**
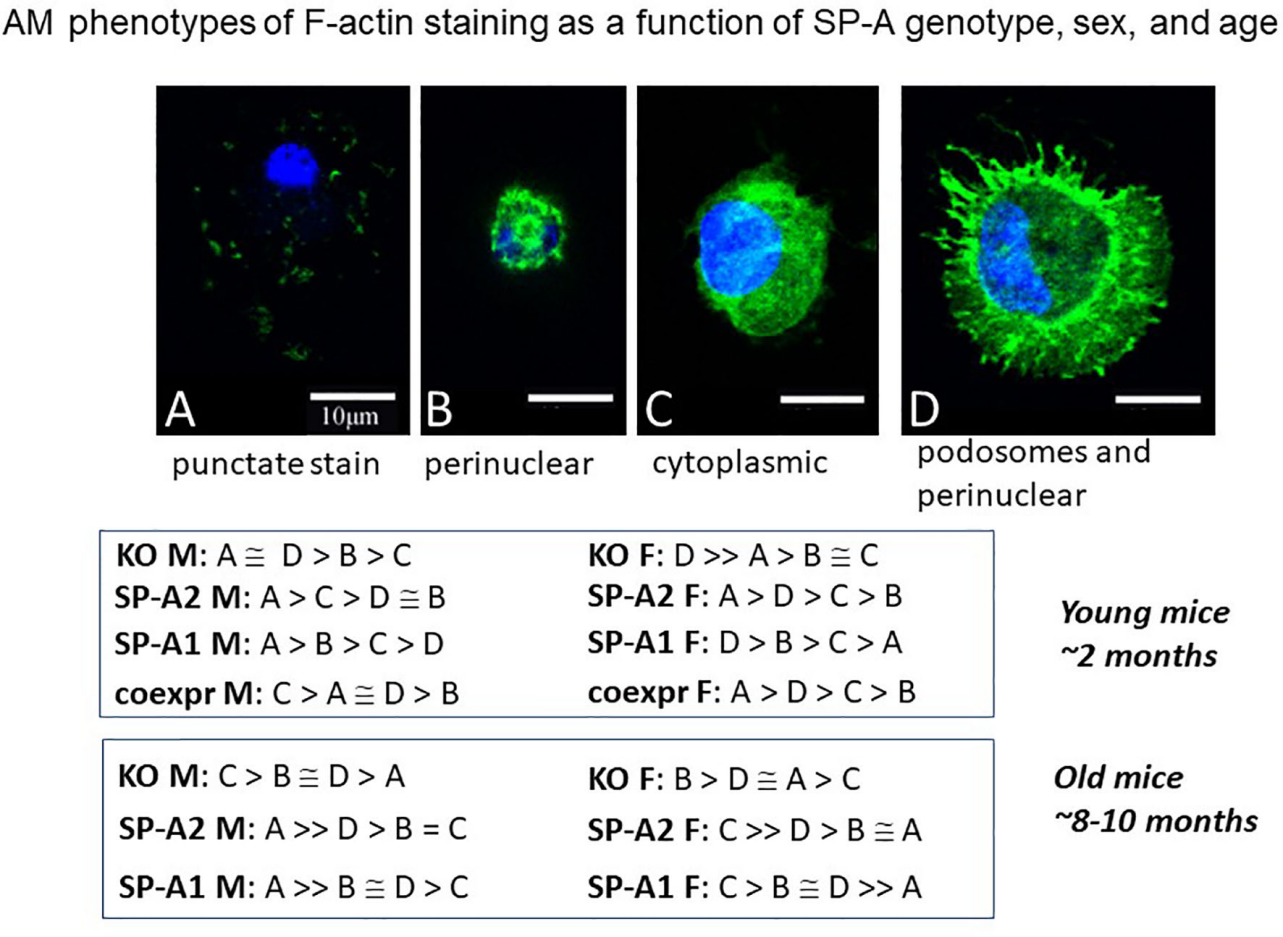
AM phenotypes of F-actin staining as a function of SP-A genotype, sex, and age. The fluorescence pattern of four types of F-actin staining is shown. The F-actin stain in **(A)** is punctate i.e. scattered cytoplasmic, in **(B)** perinuclear, in **(C)** diffuse in cytoplasm, and in **(D)** cortical with cytoplasmic protrusions (podosomes) and perinuclear. Cells were fixed, permeabilized and stained with Alexa Fluor 488-conjugated phalloidin. Images were obtained with a Leica SP8 AOBS laser scanning confocal system with software-adjusted detection spectra to avoid bleed through of the signal as described in detail by Tsotakos et al. (116). The relative frequency of these types in young and old mice are shown as a function of SP-A genotype and sex.

Another study employing an imaging approach of single cell analysis using fluorescence and confocal microscopy showed AM population heterogeneity (116). The F-actin cytoskeleton assessment, based on the fluorescence intensity of phalloidin staining, revealed a number of AM phenotypes (A-D). These phenotypic subpopulations differed in an SP-A genotype-, sex-, and age-dependent manner **(** **Figure 10** **),** as well as to whether AM were exposed to SP-A1 or SP-A2 chronically or acutely (not shown). The G-actin intensity changed among the F-actin-based AM subpopulations, and measurements indicated that these cell phenotypic responses were likely due to changes in gene expression rather than simply cytoskeleton remodeling (116). In old male SP-A1 or SP-A2 mice, the AM subpopulation associated with AM inactivation was increased, but this was not quite the case in females. In young mice the frequency of each of these actin-based phenotypes was the same in SP-A2 and co-ex females indicating that the presence of SP-A1 (in co-ex) may not have any major effect on the phenotypes compared to those from AM exposed to SP-A2 only (116). Similarities as well as differences of the impact of SP-A2 *vs* co-ex in various read-outs have been shown with the AM miRNome (117) and survival after bacterial infection (118), as well as with proteomics where the SP-A2 AM profile was most similar to wild type (that may be considered closer to co-ex), especially for actin cytoskeleton proteins and proteins regulated by Nrf2 (112). Collectively, these indicate that SP-A2 may be the driving force for the determination of AM phenotype especially in host defense processes. While there is no direct evidence of the functional significance of these subpopulations, the speculation that they reflect different stages of activation seems reasonable, and is supported by studies addressing the role of actin in polarity of macrophage function in different systems. Inhibition of ROCK kinase, a key regulator of the actin cytoskeleton, affected macrophage polarity and function (119). Importantly, G-actin was identified as a potential target of macrophage polarization in anti-tumor therapy (119).

Actin cytoskeleton dynamics are critical for the main functions of AMs, such as phagocytosis (78, 79, 85), and phenotypic differences may reflect differences in the activation status of the AMs (116). While this assumption has yet to be confirmed, a novel subpopulation of AMs (CD11c^+^ CD11b^+^) was described in the BAL of old mice. These AMs display pro-inflammatory characteristics and are permissive to *Mycobacterium tuberculosis* (120). The lung environment may control the metabolic status of these cells, thereby affecting their activation status (121). Currently it is not known whether and how SP-A1 and SP-A2 may differentially affect AM responsiveness to *M. tuberculosis*, but numerous studies (as discussed in this review) have shown that the two gene products differentially affect nearly all the AM read-outs studied to date, with SP-A2 being more effective in the enhancement of innate immune-related responses, such as pro-inflammatory responses, as well as morphological changes, as seen by the actin-related proteome differences (114).

### 6b. Alveolar Cell miRNome

SP-A1 (6A^2^) and SP-A2 (1A^0^) differentially regulate the miRNome of the epithelial Type II cell (122) and the AM (123) in a genotype-dependent and sex-specific manner at 4hr post O_3_ exposure and after normalization to the corresponding SP-A KO groups. SP-A2 had a significant impact on the AM miRNome and SP-A1 on the epithelial Type II miRNome. In both cell types the miRNome of males (but not females) changed significantly in response to O_3_ exposure. Gonadectomy had a major impact on the miRNome of males when compared to females under both control conditions (filtered air-exposed mice) and after O_3_ exposure, indicating a role of sex hormones. A considerable amount of evidence exists that sex hormones do indeed control immune response (124).

The SP-A2-mediated changes in the AM miRNome in males showed (at 4hr post O_3_ exposure) their miRNA target genes to be involved in pro-inflammatory, anti-apoptotic, and antioxidant pathways (123). But when both SP-A1 and SP-A2 gene products were expressed (co-ex) in the same humanized transgenic (hTG) mouse, both similarities and differences were observed in the specific AM miRNA-targeted genes and pathways when compared to hTG SP-A2 male mice under similar conditions (i.e., at 4hr post O_3_ exposure) (117). The common pathways included the pro-inflammatory response (STAT3, NF-kB) and the anti-apoptosis pathway (117). However, in males expressing only SP-A2, the reactive oxidant species (ROS) homeostasis pathway was involved, whereas in males expressing both SP-A1 and SP-A2 proteins the cell cycle, growth, and proliferation pathway was involved (123). A recent study where the NAD(H) redox status of AM after O_3_ exposure was investigated using optical redox imaging showed SP-A2 to be involved in processes of ROS homeostasis (103). AMs from males were more oxidized and had higher mitochondrial ROS than females. Together these indicate that AM from SP-A2 males are more sensitive/responsive to O_3_ exposure than females, and as a result there may be a more robust regulation of the AM male miRNome and its miRNA-targeted genes involved in ROS-homeostasis pathway. But when both SP-A1 and SP-A2 proteins are present perhaps a more sustained recovery is underway, as assessed by the finding where the regulated AM miRNAs and their target genes are involved in cell cycle, growth, and proliferation processes. In co-ex, although a downregulation was largely observed in the miRNome of both male and female mice, the expression of target genes studied was opposite in males (upregulation) and females (downregulation) (117). The SP-A2 males showed overall similarities with the co-ex males where there was largely a downregulation of the miRNome and upregulation of the target genes (117, 123). Moreover, differences in the AM miRNome and their target genes between co-ex and SP-A2 male and female mice persists even at 18hr post O_3_ exposure, with O_3_ exposure shown to attenuate sex differences (125). At this time point the overall pathways were similar but the direction and the number of changed miRNAs in response to O_3_ varied, indicating underlying complexities in the mechanisms involved and an interplay of O_3_ exposure, time of post exposure, sex, and SP-A genotype.

### 6c. AM Gene Expression

Differences between SP-A1 (6A^2^, 6A^4^) or SP-A2 (1A^0^, 1A^3^) variants, and KO in response to infection are observed in the regulation of AM gene expression in males and females (126). Ingenuity Pathway Analysis (IPA) revealed key pathways and molecules involved in TP53, TNF, and cell cycle signaling nodes. All variants, except SP-A2 (1A^3^) female, showed significance for at least two of the three pathways studied, and the KO male showed significance for all three pathways. The expression profile of validated genes was sex- and variant-specific, and a similarity in the gene expression profile of KO and SP-A1 mice was observed (126).

### 6d. Toponomics of Alveolar Macrophages

**Figure 11:**
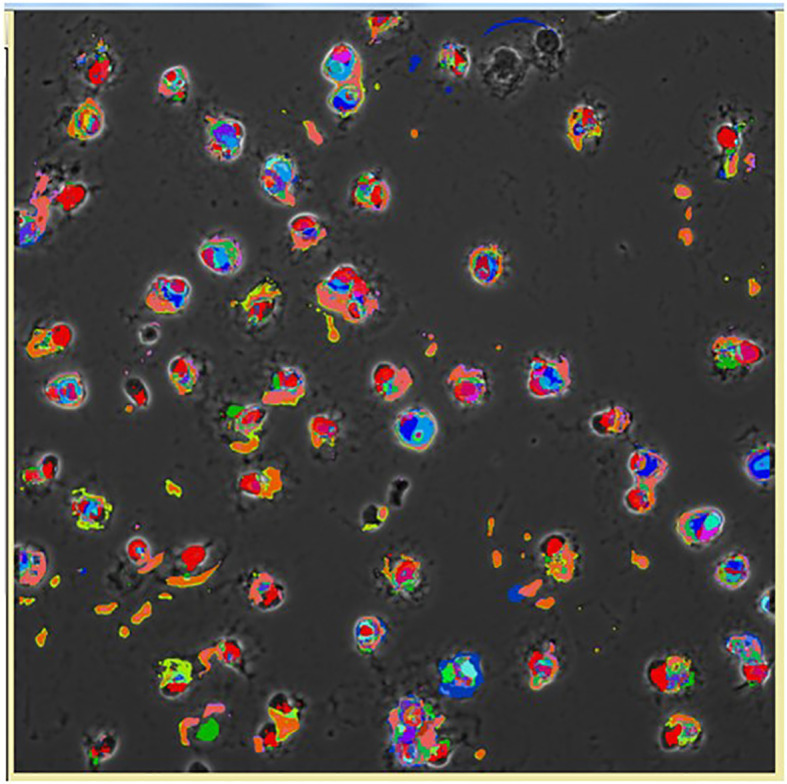
Pseudocolored image of cells based on the CMPs within each cell using the Toponome imaging system. Pseudocolors correspond to each CMP present in the given cell and are assigned by the Multicompare program. These pseudocolors are then superimposed on the appropriate phase contrast image as described in detail by Phelps et al. (132). Please note that no two cells are identical.

The presence of SP-A affects multiple functions or cellular states of the AM, as discussed above. In general, in nearly all quantitative studies, a number of cells are combined or pieces of tissue are used, so differences between individual cells are lost, and the specific read-out usually either increases or decreases or remains unchanged. However, this by itself cannot provide any insight regarding “patterns of protein combinations” in a macromolecular cluster within individual cells. The Toponome Imaging System (TIS), a serial immunostaining system combining aspects of the *proteome* and *interactome*, permits the co-localization of multiple proteins within intact single cells, and allows the identification and enumeration of supramolecular structures formed by protein clusters, known as combinatorial molecular phenotypes or CMPs (127–131). CMPs are likely the basis of cellular function because proteins usually function as part of a complex rather than alone. An example of a pseudo-colored image of cells with colors corresponding to each CMP within a given single AM is shown in **Figure 11**. This provides a visual depiction of the diversity of AM, where no two AMs are identical. AMs from SP-A1, SP-A2, or KO mice exhibit an extensive CMP-based diversity (132) that may translate into functional differences and serve as the basis of the SP-A variant-dependent effects in AMs described above, and shown previously in other systems (127, 133, 134). We found SP-A1 AMs to be more heterogeneous than either KO or SP-A2 AMs. A similar CMP-based AM heterogeneity was observed previously in AM from SP-A KO and SP-A1-rescued KO mice (135), where a single SP-A1 treatment of SP-A-KO mice increased CMP-based diversity compared to those treated with vehicle. The CMPs in KO were more conserved (i.e., present in all or most members of the experimental group) than in SP-A1 treated cells. The less abundant CMPs were more diverse i.e., these were composed of a greater number of markers. The CMP data analysis clearly showed that “patterns of protein combinations” between the two groups (presence or absence of SP-A1) are not all-or-nothing and certain CMPs are predominant in one or the other group and some are unique to one or the other group. This rheostat-like CMP presence may have functional consequences and be the basis of SP-A1- and SP-A2-mediated functional and regulatory differences observed in AMs discussed above. Our unpublished studies also show sex-dependent CMP differences in AM. Combined, these show a role for SP-A in AM diversity based on CMPs. As the proteins in a given CMP hold the potential to interact with one another to mediate or contribute to a given function, CMP differences may underlie not only the SP-A variant-mediated AM function and regulation discussed above, but other roles yet to be described.

## 7. Functional Read-Outs in the Intact Organism

### 7a. Lung Function and Survival in SP-A1 and SP-A2 hTG Mice

hTG mice, each expressing a different SP-A1 or SP-A2 variant, revealed differences in airway lung function read-outs after *K. pneumoniae* infection. In the absence of methacholine, sex differences were observed for most parameters studied, with no significant differences being observed between SP-A1 and SP-A2 variants. In the presence of methacholine both sex- and variant-dependent differences were observed (136). The SP-A2 (1A^3^) exhibited increase in the lung function read-outs in both sexes when compared to SP-A2 (1A^0^), and the SP-A1 (6A^2^, 6A^4^) showed more diverse lung functional responses.

Survival following *K. pneumoniae* infection differed among young (~12 weeks old) hTG mice expressing different SP-A gene variants. Mice expressing one or both (co-ex) SP-A transgenes had significantly increased survival rates following infection compared to KO (co-ex=SP-A2 > SP-A1 > KO) (118). Survival rate differences among SP-A1 and SP-A2 variants were observed with co-ex (6A^2^/1A^0^) and SP-A2 (1A^0^) exhibiting the best survival: co-ex (SP-A1 (6A^2^)/SP-A2 (1A^0^)) = SP-A2 (1A^0^) > SP-A2 (1A^3^) = SP-A1 (6A^2^) > SP-A1 (6A^4^) > KO (118). This order is reminiscent of the one obtained in the *ex vivo* phagocytosis assays (85) described above. In all cases (except for co-ex where there was no significant sex difference) females exhibited a better survival than males. For variants 6A^4^, 6A^2^, and 1A^3^, males had a significantly poorer survival compared to co-ex, females (6A^2^, 1A^3^), or 1A^0^ (males or females) (118). The lack of sex difference in co-ex indicates that expression of both gene products at similar levels, as it is in this experimental model, may eliminate the male survival disadvantage following bacterial infection. However, this may not be the case if the relative levels in BAL of SP-A1 and SP-A2 vary significantly. Variable levels in BAL were seen in the ratio of SP-A1 to total SP-A in healthy subjects and in various patients; the ratio of SP-A1 to total SP-A was increased in subjects with asthma (137), and in cystic fibrosis and bacteria positive samples (73). Although this ratio is not based on absolute levels due to the different affinities of the antibodies used, it is however a useful tool to indicate that differences do exist in health and disease and perhaps also as a function of age (73). The details of the SP-A1 antibody generation and specificity as well as the assays used are described by Tagaram et al. (73). Following these studies an SP-A2-specific antibody became commercially available (126). The SP-A antibody that recognizes both SP-A1 and SP-A2 was generated (87) against purified SP-A (138) from alveolar proteinosis. Future studies are needed using antibodies with equal affinities, to determine the range of absolute values in health and disease and/or among various populations.

In aged mice (~9-12 months) a significantly better survival after *K. pneumoniae* infection was observed in mice expressing the SP-A2 (1A^0^) variant with no major variant- or sex-specific differences being observed among the other variants, except for the SP-A1 (6A^2^) that showed better survival in females compared to males (139). A serendipitous and perhaps a clinically relevant observation (140) was made in the course of this survival study. Improved ventilation, as provided by the experimental conditions (i.e., high flow of humidified, warmed (37.5°C) and at constant pressure of 1.5 cm H_2_O filtered air), resulted in a significantly better survival of aged infected mice compared to those with infection alone and without prior improved ventilation; The SP-A2 (1A^0^) provided a better overall survival in both males and females. The improved survival with prior ventilation held true for young mice (~12 weeks old), but the magnitude of change was considerably smaller in young mice compared to aged mice (~9-12 months old) (139). O_3_ exposure prior to infection had an overall negative impact on the survival of aged mice compared to those with improved ventilation prior to infection. A gene-, sex-, and variant-specific survival was observed in the O_3_-exposed group that was subjected to improved ventilation prior to infection. The SP-A2 variants were associated with better survival in males and the SP-A2 (1A^0^) variant with better survival in females compared to the SP-A2 (1A^3^) variant.

The SP-A variant-, exposure-, and age-dependent survival clearly indicates that SP-A has a significant positive effect. Although a single SP-A dose led to a significantly better survival after infection, it is not known which SP-A variant (if used for therapy) could positively affect survival in the presence of additional environmental insults. Thus, it would be of interest to identify SP-A variants or functional SP-A fragments that, for example, could resist O_3_-induced oxidation and thus maintain all or most of their functional capacity under those conditions.

### 7b. Survival in Humans After Lung Transplantation as a Function of SP-A Genetics

In humans, after lung transplantation, survival was significantly better in the first year, which is the most critical time for these patients, if the SP-A2 genotype of the donor lung was 1A^0^:1A^0^ compared to the SP-A2 1A^0^:1A^1^ genotype. The SP-A2 (1A^0^), versus other SP-A2 variants, was also associated with better survival in post-transplant patients (141), as predicted by the survival of young (118) and aged (139) hTG mice carrying different SP-A1 and SP-A2 variants. Moreover, precision-cut lung slices from human donor lung following incubation with methylprednisolone, showed that lungs carrying the SP-A2 (1A^0^) variant had higher levels of SP-A compared to lungs carrying other SP-A variants (142). Low SP-A levels have been shown to associate with poor outcome and early lung transplant survival (143). Collectively, the available data indicate an important role of SP-A genetics for optimal lung health and survival in lung transplant patients.

## 8. Association of Human Surfactant Protein A Variants With Pulmonary Disease

The importance of the human SP-A genetics in human disease is underscored by the large number of association studies, where SP-A1 and SP-A2 variants or single SP-A1 and SP-A2 nucleotide polymorphisms have been shown individually or *via* interactions between or among themselves to associate with disease susceptibility (risk or protection) or disease severity. These studies have been reviewed and described in detail elsewhere (144–147). However since the publication of these review articles, the list of such studies continues to grow (95, 148–153). This is not surprising because innate host defense, regulation of inflammatory processes and/or surfactant dysregulation/derangement are central to most (if not to all) pulmonary diseases and SP-A is shown to play a role in these processes. Thus, a better understanding of the functional, biochemical, regulatory and other differences among the SP-A genetic variants in health and disease is of the utmost importance if we were to improve disease-specific and patient-specific decision making in the clinic and consider potential SP-A-specific therapies for various patient groups or individuals. The significantly improved survival of bacterial infected SP-A KO animals after a single dose of SP-A treatment (118) indicates the feasibility of such studies and as discussed in the last section of this review, efforts are underway in preclinical studies towards the use of SP-A therapeutically. Moreover, the potential role of these variants in infectious diseases such as in COVID-19 has been discussed elsewhere (154, 155).

## 9. Discussion

**Figure 12:**
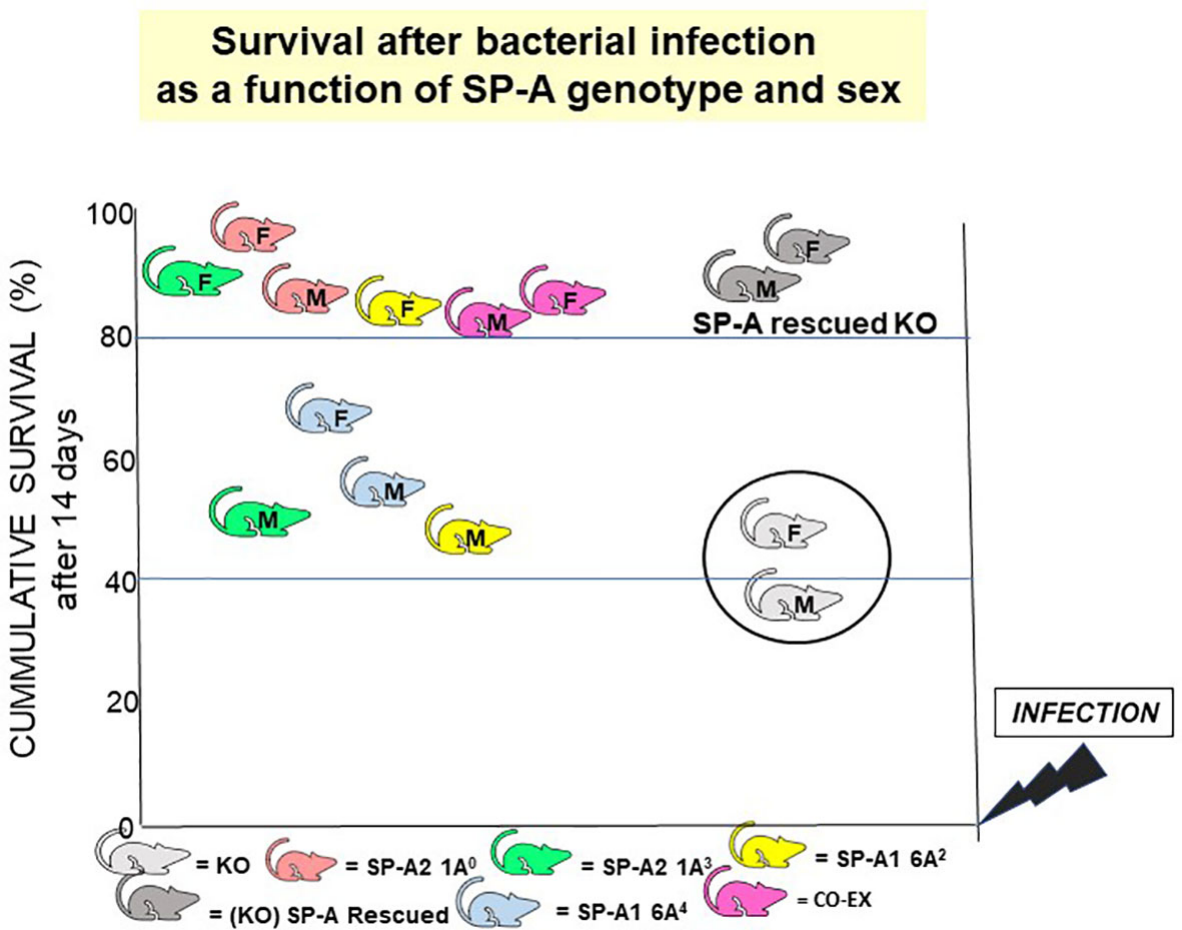
SP-A genotype- and sex-dependent survival after bacterial infection. This cartoon shows that: a) in the absence of SP-A i.e., KO, survival after *Klebsiella pneumoniae* infection is poor but survival is significantly improved if the KO mice are rescued with SP-A; and b) survival differs as a function of SP-A genotype and sex (118). However, in the absence of infection, no significant differences have been observed in mice expressing different variants or in SP-A KO mice, if the mice are housed in a pathogen-free environment.

If the gold standard for an innate immune molecule were to be survival of the organism in response to an insult (i.e., infection), then SP-A is an important host defense molecule in the lung. The survival of young SP-A KO mice after infection and/or certain processes associated with *in vivo* infection are considerably poorer compared to wild type animals (100, 156–159). Moreover, a single dose of exogenous SP-A treatment of SP-A KO mice prior to infection, after infection, or at the time of infection significantly improved survival (118). The overall survival after infection, regardless of SP-A variant, in most cases depending on the sex of the animal, is significantly higher than the KO (118), even though no significant difference is observed among the various SP-A mouse lines without infection. A cartoon depicting this SP-A variant- and sex-dependent survival outcome is shown in **Figure 12**. These observations indicate, not only that SP-A is key for effective lung innate immunity, but that the SP-A genetics variably alter outcome and thus are likely to contribute to lung disease susceptibility.

Age and O_3_ exposure prior to infection have a negative impact on survival (100, 139). Observed differences in survival among variants of infected young mice (118), get minimized or eliminated in aged infected mice after O_3_ exposure (139). Of interest, the higher survival observed with the SP-A2 (1A^0^) variant in aged mice, was also observed in human lung transplant patients who tend to be of older age (149). However, a serendipitous observation (139) revealed that high flow of humidified and warmed air significantly: a) improved survival in aged mice (and young mice but to a lesser degree); and b) in certain cases this improvement was SP-A variant- and sex-dependent. Moreover, numerous studies have revealed associations between SP-A genetics variants and lung disease susceptibility (145–148, 150, 151, 160–163) further underscoring the contribution of SP-A genetics to disease susceptibility. Together these clearly indicate a differential and a beneficial role of SP-A variants. Although the observed variant-specific differences under certain conditions may be partially obliterated, the addition of other non-invasive clinical procedures, such as high flow of humidified and warmed air, improves SP-A variant- and sex-dependent survival. Whether this is the case in humans remains to be determined (140).

**Figure 13:**
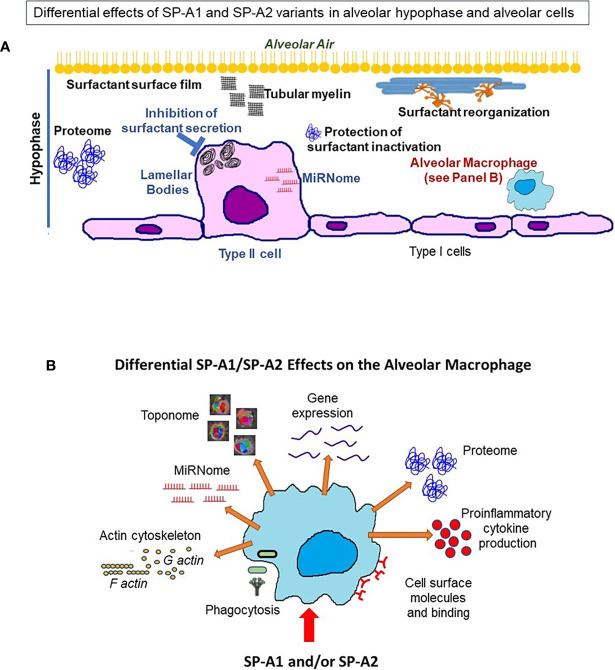
This cartoon provides an overview of the processes reviewed here to be differentially affected by SP-A1 and SP-A2 gene products in the alveolus, the BAL/hypophase and the alveolar cells (macrophages and epithelial cells). These processes are detailed in **Table 4**. **(A)** shows a cartoon of such processes of the type II cell miRNome, inhibition of surfactant secretion and perhaps the size of lamellar bodies. No studies involving SP-A1 and SP-A2 have been carried out for the type I cells. This panel also depicts events occurring in the hypophase. These include surfactant reorganization, surfactant surface film, proteome, and protection of surfactant inhibition by serum proteins. Both SP-A1 and SP-A2 are needed for tubular myelin formation. **(B)** depicts a cartoon of SP-A1 and SP-A2-mediated processes that affect AM at baseline and/or in response to insults. These include differences in AM proteome, toponome, gene expression, miRNome, bacterial phagocytosis, pro-inflammatory cytokine production, expression of cell surface molecules, AM binding and actin cytoskeleton.

The question now is what happens between infection and the ultimate differential survival of mice expressing different SP-A1 and SP-A2 variants. As reviewed here several processes and functions are differentially affected by these variants in the epithelial type II cells (no studies have been done with the type I cells), the alveolar macrophage, and the hypophase i.e., the microenvironment these cells are exposed to. A cartoon is shown in **Figure 13** depicting processes and/or functions of the alveolar type II cell, the hypophase, surfactant structures and the AM that SP-A1 and SP-A2 may differentially affect (**Table 4**). Thus, in the alveolar space, the AM, for example, as a resident cell is exposed to a different local microenvironment depending in part on SP-A genotype. At the same time the AM, and perhaps other alveolar cells, differentially contribute to the local microenvironment as a function of the SP-A variant exposure and sex. This may affect both the AM’s baseline properties and subsequently its differential response to various insults, as this may be reflected by changes in its proteome, toponome, miRNome, gene expression, or other. Rescue experiments with SP-A of SP-A KO mice support SP-A-mediated changes both at baseline conditions and in response to insults. For the former, a single dose of SP-A treatment of SP-A KO mice resulted in an AM proteome that resembled that of the WT AM and for the latter, a single dose of SP-A treatment of KO mice resulted in a better survival after infection. Moreover, SP-A genetics, as discussed, has a major regulatory impact on processes and functions of AM underscoring the importance and the complexity of the AM/SP-A interaction in health and disease.

**Table 4:** Functional differences between SP-A1 and SP-A2.

SP-A1 compared to SP-A2 exhibits more robust response or higher levels/activity.	References	
Inhibition of surfactant inactivation by serum proteins	(22)
Efficiency of surfactant reorganization	(22)
Increased alveolar macrophage heterogeneity *via* toponomics analysis	(132, 135)
Epithelial type II cells may contain a larger size of lamellar bodies	(56)
Response of the type II cell miRNome in males after mouse exposure to O_3_	(122)
Inhibitory effect on alveolar macrophage binding by SP-A2	(113)
Larger size of protein oligomers	(23)
F-actin levels in AM	(110)
**SP-A2 compared to SP-A1 exhibits more robust response or higher levels/activity.**	
Ability to bind phagocytic cells	(118)
Binding capacity to AM	(113)
Bacterial phagocytosis by alveolar macrophages	(78, 79)
Expression of AM surface molecules, CD14 and TLR-2	(118)
Phagocytic sensitivity after SP-A exposure to O_3_	(85)
Binding affinity to a wider variety of sugars	(86)
Proinflammatory cytokine production by a macrophage-like cell line	(87, 88)
Survival in young animals after bacterial infection	(118)
Survival in aged mice after infection with or without prior O_3_ exposure	(139)
Survival in lung transplant patients	(149)
Inhibition of ATP-stimulated phosphatidylcholine secretion by epithelial type II cells	(23)
Susceptibility to degradation by trypsin due to Arg85	(61)
Ability in aggregating self, DPPC and DPPG lipid vesicles, and LPS	(54, 61)
Collagen domain stability by circular dichroism and fluorescence spectroscopic studies	(54)
Response of the AM male miRNome after mouse exposure to O_3_	(123)
Response of the BAL proteome in males after infection and O_3_ exposure	(111)
**Other characteristics of SP-A1 and SP-A2**	
Similarity of the SP-A1 AM gene expression profile with that of the KO after infection	(126)
Similarity of the male SP-A2 and co-ex miRNome in response to O_3_. Ozone exposure attenuates sex-differences in the AM miRNome.	(117, 125)
SP-A2 survival is similar to co-ex	(118)
Oxidation and ROS content in AM males *vs* females after mouse exposure to O_3_	(103)
Different groups of proteins respond to *in vivo* SP-A1 and SP-A2 rescue in AM KO from male and female mice	(114)
SP-A2 generates DPPC/SP-B containing monolayers morphologically closer to those of SP-A from BAL	(72)
Both SP-A1 and SP-A2 are needed for tubular myelin formation	(56)
Residue 85 (Cys/Arg) plays a major role in the structure and function differences between SP-A1 (Cys 85) and SP-A2 (Arg 85)	(55)
The AM proteome of chronically SP-A-variant exposed mice exhibits subtle baseline differences compared to the AM KO proteome after SP-A rescue (acute treatment)	(112, 114)
The SP-A1 and SP-A2 AM proteome is similar if mice express low levels of SP-A1 and SP-A2 unlike that of high SP-A level expressors	(112)
SP-A1 and SP-A2 differentially affect actin cytoskeleton in a sex- and age-specific manner	(116)

In some cases, especially in the absence of SP-A, some unexpected and rather paradoxical findings were observed. These unexpected and paradoxical findings provided further insight into the various roles of SP-A in the alveolar space/cells, especially as one thinks of the SP-A’s “gold standard” outcome (i.e., survival of the organism after infection). Such examples include the following. The BAL proteome from SP-A KO animals exposed to filtered air was reminiscent of that from wild type animals in response to O_3_ exposure (102) indicating that in the absence of SP-A, the KO may be in a state of chronic oxidative stress. This now has been confirmed in a recent study where the redox status of KO and SP-A2 AM was assessed using a redox imaging technique and was shown to be sex-dependent (103). Also, in the same study SP-A2 in a sex-dependent manner affected the NAD(H) redox homeostasis of the AM in response to both filtered air and O_3_ exposure (males) or O_3_ exposure (females). Another interesting observation is that following analysis of the KO and WT BAL proteome after bacterial infection. Certain proteins involved in host defense were upregulated in SP-A KO BAL under baseline conditions, indicating perhaps, an attempt to overcome host defense deficits in the absence of SP-A. However, these were decreased after infection and the expression of other proteins involved in host defense were altered in KO compared to WT. These changes, because they do not, as shown by survival studies of infected mice, translate into survival rates seen in WT mice, indicate a dysregulation in one or more SP-A-mediated processes discussed in this review.

Whether the two SP-A gene products and their corresponding variants are equivalent in function, structure, or other (164), after evaluation of the collective literature, it is clear that the SP-A1 and SP-A2 gene products exhibit both unique and overlapping function/activity with extensive qualitative differences among variants. Each SP-A genotype exhibited differences from one another in nearly all the read-outs studied. **Table 4** summarizes a number of functions where one SP-A gene product exhibits a more robust response, higher functional activity, or various characteristics that differ from the other. However, SP-A2 shared more similarities with the co-ex (presence of both SP-A1 and SP-A2) in a sex-specific (or not) manner, in several of the read-outs, especially, the ones associated with or holding the potential to regulate innate immune responses. These may include studies relating to survival, AM miRNome, proteome, actin cytoskeleton, and other. In some cases, the pattern of expression or regulation as, for example, was shown for the AM proteome, actin-related pathways, and others, was opposite between SP-A1 and SP-A2. The SP-A1, on the other hand, compared to other SP-A genotypes (SP-A2, KO) exhibited more diverse patterns (i.e., less consistency/similarity). For example, in the AM gene expression pathways, SP-A1 exhibited some similarity with the KO AM, whereas with regards to the AM CMP-based diversity, SP-A1 showed more heterogeneity than either the KO or SP-A2. SP-A1 also had an inhibitory effect on AM binding by SP-A2, and a lower ability to self-aggregate by itself or in the presence of SP-A2, but a better efficiency in surfactant lipid membrane reorganization, plus a better protection against surfactant inactivation by serum proteins.

In summary, SP-A variants differentially affect the BAL proteome, and consequently the local microenvironment where the sentinel lung innate immune cell, AM, resides, and with which SP-A interacts to regulate several of its processes and functions (**Figure 13B** and **Table 4**). In other words, SP-A variants may not only differentially affect AM processes and function in response to various insults, but also the status of AM “readiness” to respond to various insults. In fact, analysis of combinational molecular phenotypes (CMPs) in individual AM cells under unprovoked conditions showed that AM exposed continually *in vivo* to different SP-A variants exhibit an extensive and variable CMP-based diversity. This CMP diversity may be the basis of SP-A variant-mediated effects observed in AM. As single CMPs or groups of CMPs may be poised to mediate a specific function in the cell, we postulate that some of the unexpected findings in SP-A KO are partly the result of the shared CMPs observed between SP-A KO and AM exposed to SP-A1 or SP-A2. However, the ultimate positive functional outcome or maximal survival can only be realized when SP-A is present to mediate the formation of specific groups of CMPs necessary for a better outcome. Moreover, SP-A variants individually may differentially affect this CMP complexity resulting in the overall SP-A gene- and variant-specific survival differences observed. Furthermore, SP-A variants have been shown to differentially regulate the miRNome of alveolar type II epithelial cells (122) and inhibition of ATP-stimulated phosphatidylcholine secretion by Type II cells (23) **(** **Figure 13A** **).** However, no other studies have been done where the impact of SP-A genetics on various aspects of the alveolar epithelial Type II or Type I cells was investigated. Thus, additional mechanisms may mediate changes in the local microenvironment that may control the metabolic status of the alveolar cells, thereby affecting their functional/activation status (121). These may involve, either SP-A variant-mediated processes not yet studied or non-SP-A mediated processes, or both.

On a final note, the use of SP-A in the clinic, and especially in the prematurely born infants who may develop respiratory distress syndrome or bronchopulmonary dysplasia has not been considered (165). These infants exhibit not only pulmonary surfactant deficiency (166), but also SP-A deficiency (67, 167, 168). Although surfactant replacement therapy is routinely used, none of the preparations contains SP-A even though a major complication in these infants is infection. Based on the available literature, inclusion of SP-A, or biologically functional fragments of SP-A in the existing surfactant preparations or SP-A usage alone must be considered for treatment of this patient population. Prematurely born infants historically constitute the first patient population where surfactant deficiency was recognized as a problem (169) and where the study of surfactant proteins began. Currently, surfactant SP-A therapy could be used, beyond the prematurely born infant, in several other diseases of infectious origin or diseases caused by exposure to various irritants/allergens. The fact that a single dose of SP-A has a major impact on animal survival and the AM proteome speak to the feasibility of SP-A therapy use in the clinic. Moreover, the potential role of the SP-A variants in infectious diseases such as COVID-19 has been recently discussed (154, 155). Furthermore, as reviewed here, SP-A may have a positive impact beyond innate immunity by benefitting surfactant-related functions, not only in prematurely born infants with neonatal disease, but in other pulmonary diseases. Of interest, trimeric fragments of human SP-A have been used successfully to neutralize RSV infection in an *in vitro* model of human bronchial epithelial cells (66). Furthermore, in animal models and human epithelial cells, exogenous SP-A is shown to attenuate asthma-related factors and protect against IL-13-induced inflammation in asthma (170).

## 10. Conclusions/Summary

The SP-A1 and SP-A2 variants, in an SP-A genotype-, sex-, age-, and condition-specific manner, differentially affect multiple read-outs in: a) the AM that may include its proteome, miRNome, gene expression, toponome, bacterial phagocytic index, cytoskeleton, and ability to produce pro-inflammatory cytokines; b) bronchoalveolar lavage (proteome); c) epithelial Type II cells (miRNome); d) surfactant structure and re-organization, and inhibition of surfactant inactivation by serum proteins; e) parameters of lung mechanics; f) survival of young and aged mice after bacterial infection with or without O_3_ exposure; and g) survival of lung transplant patients. In addition, the SP-A1 and SP-A2 variants differ in their ability to form different size oligomers and self-aggregate as well as in their sensitivity to oxidation. Thus, these differences collectively indicate that the genetics of the innate immune molecules, SP-A1 and SP-A2, and their corresponding variants, may play an important role in lung health under various conditions and this postulate is supported by the large number of studies where associations between SP-A variants and various pulmonary diseases have been observed. Furthermore, the collective evidence indicates that SP-A2 is more effective in the enhancement of innate immune-related responses and shares more similarities in several read-outs with the co-ex, where both SP-A1 and SP-A2 are expressed in the same mouse. On the other hand, SP-A1 exhibits higher efficiency in surfactant structure re-organization and in the inhibition of surfactant function by serum proteins, whereas both proteins appear to be necessary for the formation of the extracellular structural form of surfactant, tubular myelin. While the mechanisms leading to the differential effect of SP-A1 and SP-A2 on the function of AMs have not been elucidated, the initial steps may include the differential ability of SP-A1 and SP-A2 to bind to receptors in the surface of AMs. SP-A2 exhibits a higher ability to bind phagocytic cells, such as AMs and THP-1 cells (a phagocytic macrophage-like cell line) than SP-A1, but no differences were observed between SP-A1 and SP-A2 in the binding of CHO cells, a non-phagocytic cell line (118).

## Author Contributions

JF wrote most of the review, integrated all parts and figures and wrote the discussion. NTh wrote parts of the review and the Tables. NTs wrote parts of the review and contributed to some of the figures. DP wrote parts of the review and contributed to some of the figures. All authors contributed to the article and approved the submitted version.

## Funding

Supported by the CHILD Fund and the John Ardell Pursley Memorial Research Fund, Department of Pediatrics, Penn State University College of Medicine.

## Conflict of Interest

The authors declare that the research was conducted in the absence of any commercial or financial relationships that could be construed as a potential conflict of interest.

## Publisher’s Note

All claims expressed in this article are solely those of the authors and do not necessarily represent those of their affiliated organizations, or those of the publisher, the editors and the reviewers. Any product that may be evaluated in this article, or claim that may be made by its manufacturer, is not guaranteed or endorsed by the publisher.

## Abbreviations

AM, Alveolar macrophage; ARP, acute phase response; BAL, Bronchoalveolar lavage; CCT2, chaperonin subunit (beta); CMP, combinational molecular phenotypes; CRD, C-terminal carbohydrate recognition domain; DPPC, dipalmitoyl phosphatidylcholine; DPPG, dipalmitoyl phosphatidylglycerol; hTG, humanized transgenic mice; KO, Knockout; K. pneumoniae, Klebsiella pneumoniae; LPS, lipopolysaccharide; MBL3P, mannose-binding lectin family member 3 pseudogene; miRNA, micro RNA; M. tuberculosis, Mycobacterium tuberculosis; NAD(H), Nicotinamide adenine dinucleotide; NF-kB, nuclear factor kappa-light-chain-enhancer of activated B cells; Nrf2, Nuclear factor erythroid 2-related factor 2; O_3_, ozone; ROS, reactive oxygen species; *SFTPA1*, Pulmonary surfactant-associated protein A1 gene; *SFTPA2*, Pulmonary surfactant-associated protein A2 gene; SP-A, Surfactant Protein A; SP-A1, Surfactant Protein A1; SP-A2, Surfactant Protein A2; SP-B, Surfactant Protein B; SP-C, Surfactant Protein C; SP-D, Surfactant Protein D; STAT-3, Signal transducer and activator of transcription 3; *STPA3P*, surfactant protein A3 pseudogene; TIS, Toponome Imaging System; WT, wild type.
